# Hybridisation and chloroplast capture between distinct *Themeda triandra* lineages in Australia

**DOI:** 10.1111/mec.16691

**Published:** 2022-09-27

**Authors:** Luke T. Dunning, Jill K. Olofsson, Alexander S. T. Papadopulos, Samuel G. S. Hibdige, Oriane Hidalgo, Ilia J. Leitch, Paulo C. Baleeiro, Sinethemba Ntshangase, Nigel Barker, Richard W. Jobson

**Affiliations:** ^1^ Ecology and Evolutionary Biology, School of Biosciences University of Sheffield Sheffield UK; ^2^ Section for Forest, Nature and Biomass, Department of Geosciences and Natural Resource Management University of Copenhagen Frederiksberg C Denmark; ^3^ Molecular Ecology and Evolution Bangor University, Environment Centre for Wales Bangor UK; ^4^ Royal Botanic Gardens Surrey UK; ^5^ Institut Botànic de Barcelona (IBB), CSIC‐Ajuntament de Barcelona Barcelona Spain; ^6^ Department of Biological Science The University of Queensland St Lucia Queensland Australia; ^7^ Department of Plant and Soil Sciences University of Pretoria Hatfield South Africa; ^8^ National Herbarium of New South Wales, Australian Institute of Botanical Science Sydney New South Wales Australia

**Keywords:** adaptation, andropogoneae, angiosperms, ecological genetics, phylogeography, population genetics

## Abstract

Ecotypes are distinct populations within a species that are adapted to specific environmental conditions. Understanding how these ecotypes become established, and how they interact when reunited, is fundamental to elucidating how ecological adaptations are maintained. This study focuses on *Themeda triandra*, a dominant grassland species across Asia, Africa and Australia. It is the most widespread plant in Australia, where it has distinct ecotypes that are usually restricted to either wetter and cooler coastal regions or the drier and hotter interior. We generate a reference genome for *T. triandra* and use whole genome sequencing for over 80 *Themeda* accessions to reconstruct the evolutionary history of *T. triandra* and related taxa. Organelle phylogenies confirm that Australia was colonized by *T. triandra* twice, with the division between ecotypes predating their arrival in Australia. The nuclear genome provides evidence of differences in the dominant ploidal level and gene‐flow among the ecotypes. In northern Queensland there appears to be a hybrid zone between ecotypes with admixed nuclear genomes and shared chloroplast haplotypes. Conversely, in the cracking claypans of Western Australia, there is cytonuclear discordance with individuals possessing the coastal chloroplast and interior clade nuclear genome. This chloroplast capture is potentially a result of adaptive introgression, with selection detected in the *rpoC2* gene which is associated with water use efficiency. The reason that *T. triandra* is the most widespread plant in Australia appears to be a result of distinct ecotypic genetic variation and genome duplication, with the importance of each depending on the geographic scale considered.

## 1 INTRODUCTION

Understanding why some species are found in a broad range of environments whilst others are restricted to particular habitats is one of the fundamental questions in evolutionary biology. Widely distributed species are typically able to tolerate multiple environmental conditions, through a combination of extensive phenotypic plasticity and/or locally adapted genetic variation (Stamp & Hadfield, 2020). Populations that are genetically adapted to their local environmental conditions can be defined as ecotypes (Hufford & Mazer, 2003). A single species can be composed of multiple genetically distinct and locally adapted ecotypes (Linhart & Grant, 1996). It is increasingly clear that ecotypes can evolve multiple times in response to the same selection pressures (Butlin et al., 2014; James et al., 2021; Jones et al., 2012; Ostevik et al., 2012), and that this can happen over very short timescales (e.g. <250 generations, Papadopulos et al., 2021). The formation of distinct ecotypes is dependent on numerous factors including the levels of gene flow, the extent of standing genetic variation, mutation rate and the scale of environmental heterogeneity (Via & Lande, 1985). Determining how ecotypes evolve and interact when they come into secondary contact and hybridize is important for our understanding of how local adaptation can ultimately lead to speciation (Lowry, 2012; Nosil, 2012).

The origin of ecotypes in plants is often driven by the difference in soil water availability between habitats, with ecotypes from drier environments generally being smaller and flowering earlier (Latta et al., 2007; Lowry, 2012; Milano et al., 2016). Detailed studies in *Panicum virgatum* have shown that the genetic basis of ecotype differentiation is controlled by multiple loci distributed across the genome (Milano et al., 2016), and occasional gene flow among ecotypes can accelerate climate adaptation through the introgression of adaptive loci (Lovell et al., 2021). Polyploidy can accelerate ecotype divergence as relaxed selection on individual subgenomes can facilitate the accumulation of adaptive genetic variation enabling neo‐ or subfunctionalisation (Lovell et al., 2021). Genome duplication itself can also have immediate effects, with autopolyploids having increased cell size which can translate into larger plants that are more robust and produce more seed (Garbutt & Bazzaz, 1983), and which are potentially more invasive (Te Beest et al., 2012). Polyploidy can also increase reproductive isolation among ecotypes, reducing gene flow when they come into secondary contact so that they maintain their divergence (Olofsson et al., 2021). Additional work is required to determine the importance of ploidy in the formation and maintenance of ecotypes, with its role likely to be linked to population demographics and the evolutionary time over which the ecotypes have been isolated.

In plants, the chloroplast is responsible for photosynthetic energy production and optimizing its function for local environmental conditions is essential to maximize fitness. As a result, the encoded enzymes are often subject to positive selection (Hu et al., 2015; Gao et al., 2019; Yang et al., 2021). The organelles can be introgressed between species or ecotypes, in a process termed chloroplast capture. The transfer of a resident chloroplast could provide an evolutionary advantage for a species colonizing a new habitat by effectively accelerating the process of local adaptation (Muir & Filatov, 2007; Percy et al., 2014; Tsitrone et al., 2003). Chloroplast capture is typically inferred phylogenetically as a result of incongruence between the chloroplast and nuclear genome topologies that can be explained by introgression. This process has been widely documented in families across the plant kingdom (Rieseberg & Soltis, 1991). However, many of the earlier cases of chloroplast capture were identified using individual markers and subsequent reanalysis with whole chloroplast genome data has not always confirmed these cases, as seen in willows (Percy et al., 2014; Wagner et al., 2021). It is therefore essential to use whole organelle sequences to reliably infer chloroplast capture, and test for signatures of positive selection to confirm if the acquisition was adaptive rather than the result of neutral processes (Bock et al., 2014).

*Themeda triandra* is one of the most widely distributed C_4_ grass species in the world (Sage, 2017). It has a relatively recent evolutionary history, originating c. 1.5 milliion years ago (Ma), most likely in Asia (Dunning et al., 2017). *Themeda triandra* has rapidly spread across Asia, Africa and Australia where it is found across a range of climates (Snyman et al., 2013). It is a perennial tussock‐forming grass that is capable of reproducing both sexually and asexually through apomixis (Evans & Knox, 1969). Sexual reproduction is more common in diploid populations and plants are self compatible (Hayman, 1960; Liebenberg, 1990), although the actual rates of selfing are still unknown (Ahrens et al., 2020). It is a dominant grassland species with significant ecological, cultural and economic importance (Snyman et al., 2013). The great morphological diversity displayed by *T. triandra* has led to the description of different taxa with several potential synonyms (Arthan et al., 2021), ranging from regional varieties with restricted distributions to the globally invasive *T. quadrivalvis* (Arthan et al., 2021).

In Australia, *T. triandra* is the most widely distributed plant species (Gallagher, 2016) and ecotypic differences (coastal versus inland form) appear to be largely a result of variation in ploidy level (Hayman, 1960). Coastal populations that grow in cooler and wetter climates are predominantly diploid, while inland populations from drier xeric areas are prominently tetraploid (Godfree et al., 2017). Experimental manipulations of both ploidal levels collected from the same regions showed that under drought and heat stress the tetraploids produced up to four times as much seed as the diploids (Godfree et al., 2017), with elevated fitness attributed to its increased genome size. The repeated formation of polyploids within a population was thought to be the most parsimonious explanation for the origin of the tetraploids (Hayman, 1960), although a single polyploidisation event followed by the spread of that cytotype could not be ruled out (Godfree et al., 2017). This conclusion has been supported by work that shows polymorphisms within a population are common, and that tetraploids do not form their own distinct genomic group (Ahrens et al., 2020). However, both of these recent studies are limited to south‐eastern Australia and their conclusions assume that the widespread inland tetraploid ecotype is derived from the coastal diploid form, and that the ecological differences between ploidy levels are not more ancient, predating the colonization of Australia. Recent phylogenetic studies have highlighted that Australia may have been colonized by *T. triandra* multiple times (Dunning et al., 2017), but it is not yet clear whether these colonisations correspond to the different coastal and inland ecotypes.

In this study we use whole genome sequencing data from over 80 *Themeda* accessions to reconstruct the evolutionary history of *Themeda triandra* in Australia. We specifically aim to test whether the two Australian ecotypes evolved rapidly following colonization, potentially due to auto‐polyploidisation, or alternatively, if the ecotypes arrived in Australia independently. Overall, our results show that considering a broader evolutionary history rather than focusing purely on local diversity is essential to elucidate the mechanisms of rapid environmental adaptation in widely distributed species.

## 2 MATERIALS AND METHODS

### 2.1 Reference genome assembly

A de novo reference genome assembly was generated for a *Themeda triandra* accession (TtPh16‐4) collected in 2016 from the Carranglan region of the Philippines (15°56′35.8 ″N 121°00′26.2 ″E). A PacBio library was prepared by The University of Sheffield Molecular Ecology Laboratory, and sequenced on two PacBio Sequel SMRT cells. The PacBio data were cleaned and assembled using Canu version 2.0 (Koren et al., 2017) with default parameters. Organelle genomes were then generated for the TtPh16‐4 accession. The chloroplast genome was assembled using a genome walking approach (see below for details). The mitochondrial genome was manually assembled from the PacBio contigs. In brief, the complete set of mitochondrial genes was extracted from a mitochondrial assembly (NC_008360.1) of *Sorghum bicolor*, a closely related grass from the same tribe (Andropogoneae), and used as a Blastn version 2.8.1 query to identify the top‐hit TtPh16‐4 contig for each gene. These contigs were then truncated to the matching regions, retaining the intergenic regions if multiple loci were present on a single contig. Finally, duplicated regions were removed and the remaining contigs concatenated into a single pseudomolecule with gaps represented by 100 Ns. The completeness of the TtPh16‐4 mitochondrial genome was estimated using the MITOFY version 1.3.1 webserver (Alverson et al., 2010).

The TtPh16‐4 organelle genomes were used to mask organellar DNA in the Canu genome assembly prior to additional homology‐based scaffolding. Contigs containing organellar DNA were first identified using Blastn, with a minimum alignment length of 1000 bp and sequence similarity ≥99%. These scaffolds were then masked using RepeatMasker version 4.0.6 (Smit et al., 2013) with the organelle sequences as a custom database. The organelle masked contigs were then scaffolded in relation to the genome of *Sorghum bicolor* (GenBank accession: GCA_000003195.3; McCormick et al., 2018) using RagTag version 2.1.0 (Alonge et al., 2021). The TtPh16‐4 genome assembly completeness was estimated using BUSCO version 3.1.0 (Simão et al., 2015) with the poales_odb10 database, and by comparing the assembly size to the 1C genome size estimated for another individual collected from the same area (TtPh16‐2) that was estimated by flow cytometry using the one‐step protocol (Doležel et al., 2007) with minor modifications (see Clark et al., 2016).

### 2.2 Sampling and whole‐genome re‐sequencing

An initial Australia wide survey was conducted using two regions of the ribosomal DNA (rDNA), ETS and a second portion containing ITS1, 5.8S and ITS2. These data were used to assess the diversity within the T. triandra/*T. quadrivalvis* clade. Our ITS/ETS data set contained 373 ingroup accessions from across the entire *T. triandra* range (Figure S1A), including 33 previously published accessions (Dunning et al., 2017), and 340 newly sequenced accessions (271 from across Australia [Figure S1B], and 58 and 11 from across Africa and Asia respectively). We extracted DNA, amplified the target region and generated a maximum parsimony phylogenetic tree as in Jobson et al. (2017). From these data we identified 12 weakly resolved clades (Figure S1A) and selected 61 Australian *T. triandra* samples for whole genome resequencing to represent the geographic range within each. We also selected six outgroup samples for sequencing, including three Australian *T. quadrivalvis*, two *Themeda avenacea* and a single *Themeda arguens*. The 67 selected *Themeda* accessions were then sent for sequencing at the Ramaciotti Centre for Genomics (Sydney, Australia). Libraries were constructed using the Illumina DNA Prep kit and sequenced on an Illumina NovaSeq 6000 with the aim of generating 10 Gb of 2 × 150 bp data per sample. Short‐read Illumina data was also generated for the TtPh16‐4 Filipino accession, with Illumina DNA Prep libraries constructed at the Sheffield Diagnostic Genetics Service (UK) and sequenced on a full lane of an Illumina HiSeq2500. Estimated genome coverage for each sample was calculated using the TtPh16‐2 accession 1C‐value.

### 2.3 Chloroplast genome assembly and phylogenetics

Chloroplast genomes were assembled from the raw whole genome sequencing data using NOVOPlasty version 4.2.1 (Dierckxsens et al., 2017) with default parameters and a *matK* seed alignment extracted from a chloroplast genome assembly of a closely related species (Andropogoneae: *Heteropogon* sp., GenBank accession: KY707768.1). The resulting contig options were aligned to pre‐existing *T. triandra* and *T. quadrivalvis* chloroplast assemblies from NCBI genbank using MAFFT version 7017 (Katoh et al., 2002), and manually rearranged so that the short single copy and inverted repeat was in the same orientation for each individual using Geneious version 5.3.6 (Kearse et al., 2012) if required. If the chloroplast assembly was incomplete, the process was repeated using a different seed alignment (the *rbcL* gene or the entire chloroplast sequence from the same *Heteropogon* sp. accession). Phylogenies were inferred with and without one of the inverted repeats. The inverted repeats within a plastome recombine with each other meaning they are generally identical and including them effectively inflates the weight to these positions (Blowers et al., 1989; Palmer, 1985). Maximum‐likelihood phylogenetic trees with 100 bootstrap replicates were inferred using PhyML version 20120412 (Guindon et al., 2010), with the best‐fit nucleotide substitution model selected with SMS version 1.8.1 (Lefort et al., 2017).

### 2.4 Mitochondrial genome assembly and phylogenetics

We used a reference‐based approach to generate consensus sequences for the mitochondrial genome (Bianconi et al., 2020). The 68 *Themeda* samples sequenced here (the 67 samples selected across the Australian *T. triandra* range plus TtPh16‐4) were supplemented with 14 *T. triandra* and *T. quadrivalvis* data sets retrieved from the NCBI Sequence Read Archive (SRA; Burke et al., 2016; Dunning et al., 2017; Arthan et al., 2021). Prior to mapping the *Themeda* sequencing data to the TtPh16‐4 genome, the data were cleaned using Trimmomatic version 0.38 (Bolger et al., 2014) to remove adaptor contamination, low quality bases (4 bp sliding window with mean Phred score <20) and short reads (<50 bp). NGSQC Toolkit version 2.3.3 (Patel & Jain, 2012) was then used to discard reads where 80% of the sequence had a Phred score <20 or the read contained an ambiguous base. Finally, PRINSEQ version 0.20.3 (Schmieder & Edwards, 2011) was used to remove duplicated reads. The cleaned data were then mapped to the TtPh16‐4 reference genome using Bowtie2 version 2.3.4.3 (Langmead & Salzberg, 2012) with default parameters. Consensus sequences for each *Themeda* sample were generated from the short‐read alignments to the mitochondria using previously described methods (Bianconi et al., 2020). In short, only bases with five times the expected nuclear genome coverage were called to remove the potential effect of organelle‐nuclear transfers. The alignment was subsequently trimmed with trimAl version 1.2rev59 (Capella‐Gutiérrez et al., 2009) using the ‐automated1 option which optimizes alignment trimming for maximum‐likelihood phylogenetic tree reconstruction. A maximum‐likelihood tree was inferred from the 429,412 bp alignment using IQ‐TREE version 1.6.12 (Nguyen et al., 2015) with 1000 ultrafast bootstrap replicates.

### 2.5 Nuclear marker assembly and phylogenetics

We inferred the nuclear history of *Themeda* using two different data sets, the nuclear ribosomal DNA (rDNA) array and genome‐wide single‐copy loci. We assembled the rDNA array using the same method as Becher et al. (2020). This approach consisted of using NOVOPlasty with a 594 bp reference rDNA sequence for the seed alignment from a closely related species (Andropogoneae: *Heteropogon triticeus*, GenBank accession: KY991073.1). The resulting assemblies were processed in the same way as the chloroplast (see above), and the alignment was subsequently trimmed to the 5.80 kb rDNA coding region consisting of 18S, ITS1, 5.8S, ITS2, 26S. A phylogenetic tree with 100 bootstrap replicates was inferred using PhyML, with the best‐fit nucleotide substitution model selected with SMS.

A reference‐based approach was used to generate consensus sequences for single‐copy nuclear genes to infer phylogenetic relationships (Dunning et al., 2019; Olofsson et al., 2016, 2019). Our analysis focused on the 3303 Benchmarking universal single‐copy orthologues (BUSCO; Simão et al., 2015) in the poales_odb10 database identified in the TtPh16‐4 genome. Consensus sequences for each *Themeda* sample were then generated from the bowtie2 alignments generated above for each single‐copy gene in the TtPh16‐4 genome using previously described methods for low‐coverage whole genome data (Dunning et al., 2019; Olofsson et al., 2016, 2019). Each individual gene alignment was subsequently trimmed with trimAl, short sequences (<200 bp) were discarded from the trimmed alignment, and the entire gene alignment was discarded if it was either <500 bp or did not include all samples (*n* = 2096 genes retained).

A maximum‐likelihood tree was inferred from a concatenated nuclear alignment of all 2096 genes (alignment length = 3,393,588 bp) using IQ‐TREE version 1.6.12 (Nguyen et al., 2015) with 1000 ultrafast bootstrap replicates. Individual gene trees were also generated using SMS and PhyML as described above. A DensiTree version 2.2.7 (Bouckaert, 2010) plot was made by overlaying the individual gene trees that could be transformed to be ultrametric using the chronopl function (lambda = 1) as part of the ape version 5.2 (Paradis & Schliep, 2019) package in R version 3.4.3. A coalescence species tree was generated from the individual gene trees using ASTRAL version 5.7.5 (Zhang et al., 2018) after collapsing branches with <10% bootstrap support using Newick utilities version 1.6 (Junier & Zdobnov, 2010). Phyparts version 0.0.1 (Smith et al., 2015) was used to evaluate individual gene tree support for the coalescence species tree. The results were visualized using the phypartspiecharts.py python script written by M. Johnson (available from: https://github.com/mossmatters/phyloscripts/blob/master/phypartspiecharts).

### 2.6 Population structure

Genotype likelihoods were estimated across the entire TtPh16‐4 nuclear genome for the 81 *Themeda* acessions (68 sequenced here) using ANGSD version 0.929‐13‐gb5c4df3 (Korneliussen et al., 2014) and the bowtie2 alignments generated above with organelle sequences excluded. Mapped reads and bases with a Phred score <20 were discarded, a per‐individual maximum depth of 20 was used, and sites had to be present in at least two individuals to be considered. A principal component analysis (PCA) of the genotype likelihoods was generated using PCAngsd version 0.973 (Meisner & Albrechtsen, 2018) to estimate a covariance matrix before plotting the results in R with eigenvector decomposition. The number of genetic clusters (*K*) in the genotype likelihoods was examined using NGSadmix (Skotte et al., 2013), with default parameters and 10 replicates for *K* between 1 and 10. The optimal *K* was determined using the Δ*K* method (Evanno et al., 2005) implemented using CLUMPAK (Kopelman et al., 2015). PCA plots and the admixture analysis were repeated for only the Australian *T. triandra* samples using default parameters. Pairwise *F*_ST_ was estimated using ANGSD among Australian populations both globally and using a sliding window method (window = 50 kb, slide = 10 kb), with this analysis restricted to individuals that had 99% of their genome assigned to a single genetic cluster.

To support the population structure results, we specifically tested for introgression between accessions by calculating Patterson's *D‐statistic* (ABBA‐BABA statistic) and the f4‐ratio using Dsuite version 0.4.r38 (Malinsky et al., 2021). To calculate these statistics we used the VCF file previously generated by ANGD and selected the combination of taxa depending on the scenario being investigated. The analysis was restricted to individuals assigned as diploid (see below), and in certain cases to those with known collection locations if geographic distance within Australia was deemed as important (Table S1). All *p*‐values were Benjamini‐Hochberg corrected to account for multiple testing and summary statistics are based solely on the significant results (corrected *p*‐value < .05). Overall, three scenarios were tested to determine if there was gene flow between: (1) *T. triandra* and *T. quadrivalvis* (outgroup *T. arguens*), (2) Asian and Australian *T. triandra* (outgroup *T. triandra* from Yemen), and (3) among Australian *T. triandra* lineages (outgroup *T. triandra* from Yemen).

### 2.7 Estimating ploidy

The ploidy of each sample was estimated using HMMploidy (Soraggi et al., 2021), a method which has been developed to infer ploidy from low‐depth sequencing data. HMMploidy uses both sequencing depth and genotype likelihoods to infer ploidy, leveraging population frequencies to account for genotype uncertainty in low‐coverage data (Soraggi et al., 2021). A multisample mpileup file was generated for HMMploidy from the bowtie2 alignments with SAMtools version 1.9 (Li et al., 2009), only including reads with a minimum read mapping quality (mapQ) of 20, counting anomalous read pairs and setting a maximum per‐file depth of 100. Genotype likelihoods were generated using HMMploidy with default parameters, which calculates likelihoods for a range of ploidy levels (up to 6x). Ploidy levels were then inferred in 100 kb windows across the chromosomes from the TtPh16‐4 reference genome, with a minimum number of two individuals per locus to be considered. The percentage of 100 kb windows supporting each ploidy level was then calculated, ignoring those that were inferred to be haploid. Accessions were arbitrarily assigned to a single ploidy if it was supported by ≥60% of windows. If no single ploidy level was supported, but the possible polyploid levels had a combined support of ≥60% of windows, then the sample was generically assigned as polyploid. Finally, if neither of these criteria were met the sample was classified as unknown ploidy.

### 2.8 Inferring positive selection

**FIGURE 1:**
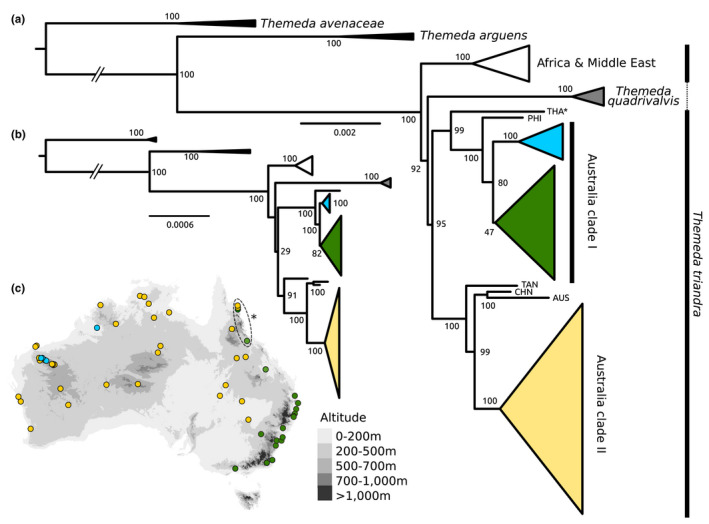
Phylogenetic relationships of *Themeda* inferred from whole (a) chloroplast (inverted repeat removed) and (b) mitochondrial genomes. In both cases, the maximum likelihood topology with bootstrap support values are shown, inferred with the (a) GTR + I + G and (b) TVM + F + R2 substitution model. For samples not assigned to a clade, a three letter abbreviation is used (THA, Thailand; PHI, Philippines; TAN, Tanzania; CHN, China; AUS, Australia). The asterisks indicate samples for which only chloroplast genomes are available. Truncated branches are indicated. (c) the sampling locations of *T. triandra* accessions in Australia clade I and II are shown, with potential hybrids from northern Queensland indicated.

Pairwise estimation of the ratio (ω) of synonymous (dS) and nonsynonymous (dN) substitutions between sequences was calculated using the Yang and Nielsen (2000) method implemented in yn00, distributed as part of the paml version 4.9j package (Yang, 2007). Site (M1a and M2a) and branch‐site models (BSA and BSA1) models, to infer if a gene was evolving under significant positive selection, were implemented in codeml, distributed as part of the paml version 4.9j package (Yang, 2007). For these models we used the topology of the whole chloroplast genome tree (Figure 1 and Figure S2).

## 3 RESULTS

### 3.1 Reference genome statistics

We generated 20.93 Gb of PacBio subread data for the TtPh16‐4 accession with an N50 read length of 5.61 kb. The initial Canu assembly was 0.70 Gb in length and consisted of 61,884 contigs with an N50 of 13.44 kb. We masked 3.08 Mb of organellar DNA before the final homology‐based scaffolding in relation to the *S. bicolor* genome. In total, 19,639 contigs were scaffolded into 10 pseudochromosomes which had a combined length of 288.99 Mb (range 21.08–46.11 Mb). We then removed scaffolds/contigs with <500 bp of sequence information. The final genome assembly was composed of the 10 pseudochromosomes, the 42,243 unplaced contigs and the organelle genomes. In total, there were 42,255 sequences, the N50 was 22.45 kb and the assembly size was 0.71 Gb (84.52% of the 0.84 Gb 1C flow cytometry estimate genome size for TtPh16‐2). The BUSCO poales_odb10 database contains 4986 genes, of which 81.5% were complete in the TtPh16‐4 genome (14.0% duplication, 2.4% fragmented and 16.1% missing). No direct genome size or chromosome count is available for TtPh16‐4, but we assume it to be diploid given the genome assembly shows relatively low levels of duplication (14.0% according to BUSCO), HMMploidy inferring the sample to be diploid, and the assembly size being smaller than the 0.84 Gb 1C flow‐cytometry genome size estimate for another accession from the same population, TtPh16‐2. Furthermore, the 1C value of TtPh16‐2 is approximately half that of previously studied tetraploids (Estep et al., 2014; Godfree et al., 2017), which also suggests TtPh16‐2 is diploid.

### 3.2 Phylogenetic relationships inferred from the chloroplast

As part of this study, we generated over 700 Gb (mean = 5.18 Gb, SD = 4.72 Gb per sample) of whole‐genome resequencing data for 68 *Themeda* accessions, with an emphasis on sampling *T. triandra* (and potential taxonomic synonyms) from across its range in Australia (*n* = 61; Figure 1). These data were used to assemble whole chloroplast genomes, before being aligned with previously published assemblies (*n* = 15). Inferring the phylogenetic relationships based on the chloroplast genomes with the inverted repeat region removed (83 accessions in total, 118,234 bp alignment, 95.4% identical sites, 97.4% within *T. triandra*; 98.6% and 99.1% when excluding indels) shows that *T. triandra* is not monophyletic, with *T. quadrivalvis* nested within. The phylogeny also shows that the Australian samples do not form a single clade (Figure 1), as would be expected if there was a single colonization of Australia. The two large Australian clades (Clade I and II) are well supported, each sister to Asian accessions. Clade I comprises the coastal *T. triandra* accessions predominantly from wetter environments (in green) and those from the Pilbara cracking claypans in Western Australia (in blue; Figure 1). Clade II is composed of the western *T. triandra* form (in yellow), which are predominantly from dryer habitats in the Australian interior. When comparing the coastal (green) and claypan (blue) *T. triandra* accessions from Clade I with the western Australia form from Clade II (yellow), there are 48 and 64 fixed biallelic SNPs respectively. Within Clade I there are 13 fixed biallelic SNPs separating the coastal (green) and Pilbara (blue) *T. triandra* accessions. The effect of retaining the inverted repeat during phylogenetic inference had very little impact on the overall tree topology (Figures S2 and S3).

### 3.3 Phylogenetic relationships inferred from the mitochondrial genome

The reference mitochondrial genome was 445,516 bp in length and consisted of four contigs joined into a single pseudomolecule. The mitochondrial genome contained the full set of expected protein coding genes, RNAs and all except two tRNAs (Arg and Gly). The mitochondrial sequences for the other accessions were obtained using a reference‐based alignment approach. After trimming, the alignment was 429,412 bp in length with a mean of 369 kb per sample (SD = 50 kb) and 99.3% of sites were identical (99.6% with *T. triandra*). The higher‐order topology was identical for both organelles (Figure 1), although there were differences within each of the clades themselves (Figures S2–S4). When comparing the coastal (green) and claypan (blue) *T. triandra* accessions from Clade I with the western Australia form from Clade II (yellow), there are 244 and 256 fixed biallelic SNPs respectively. Within Clade I there are 10 fixed biallelic SNPs separating the coastal (green) and Pilbara (blue) *T. triandra* accessions.

### 3.4 Phylogenetic relationships inferred from the nuclear genome

**FIGURE 2:**
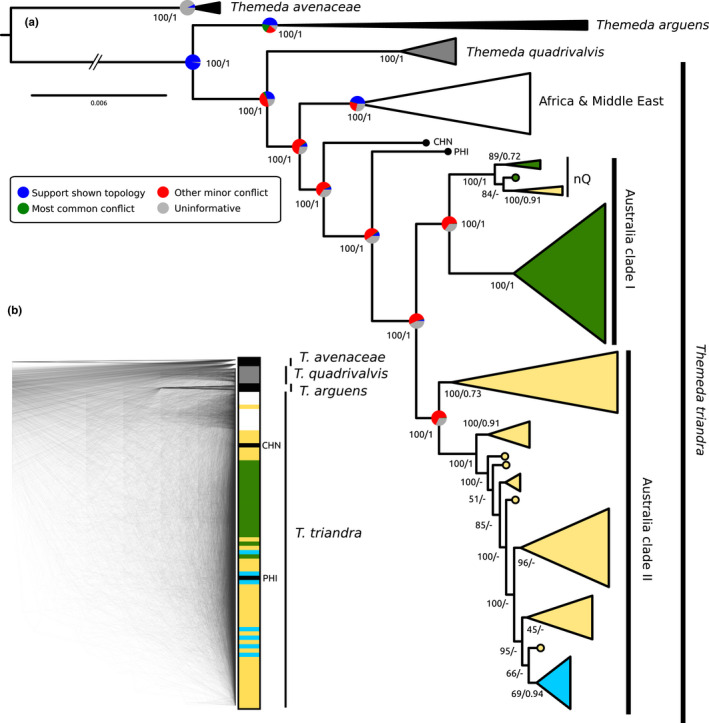
Phylogenetic relationships of *Themeda* inferred from 2096 nuclear genes. (a) a maximum likelihood topology from a concatenated alignment of all loci with colours of the Australian clades based on chloroplast groupings (Figure 1). Bootstrap support values for the concatenated maximum likelihood tree are shown, followed by local posterior probabilities from a coalescence species tree for the same nuclear loci. The pie charts on key nodes represent the individual gene tree support for the topology shown. (b) a densitree plot of overlaid nuclear gene trees. Colours match the chloroplast clades, and truncated branches are indicated. For samples not assigned to a clade a three letter abbreviation is used (PHI = Philippines and CHN = China), and the clade containing potential hybrids from northern Queensland (nQ) is indicated.

The nuclear data set was generated by mapping the short‐read data from 82 *Themeda* accessions to the TtPh16‐4 reference, with a mean of 67.5% (SD = 9.6%) of data mapping per sample. As expected, a higher proportion of data mapped for *T. triandra* (69.9%; SD = 5.6%) than for the other species: *T. quadrivalvis* (54.2%; SD = 5.5%), *T. arguens* (46.5%; SD = 3.5%) and *T. avenaceae* (28.4%; SD = 1.3%). The nuclear phylogenetic relationships were inferred using data from 2096 single‐copy genes with a combined alignment length of 3,393,588 bp. The concatenated maximum‐likelihood tree and the coalescent species tree both support *T. triandra* as being monophyletic, sister to *T. quadrivalvis* (Figure 2 and Figures S5–S7). In the nuclear phylogeny the Australian *T. triandra* accessions are monophyletic, and within this group the two distinct clades identified in the chloroplast phylogeny are recapitulated with just a few exceptions. Most notably is the Pilbara clade which is identified as Clade I based on the chloroplast data (in blue; Figure 1), but is nested deep within Clade II based on the nuclear genome (Figure 2). There are also two accessions from Far North Queensland which are part of Clade II in the chloroplast genome analyses but are nested within Clade I based on the nuclear genome data, where they form a clade with other accessions from northern Queensland. Evaluating the individual gene tree support for the nuclear topology shows that many of the nodes are poorly supported beyond delimiting the main clades, with frequent minor conflicts (Figure 2b and Figure S5). This lack of gene‐tree support for the species tree topology can be a result of incomplete lineage sorting, hybridisation and/or a lack of genetic variation.

The rDNA coding region alignment was 5.80 kb in length and 92.7% of sites were identical (94.3% within *T. triandra*). Although the rDNA tree is poorly resolved (Figure S8), several of the associations identified using the single copy nuclear loci are also recovered (Figure 2), namely the association of the Pilbara accessions (blue) with those from Clade II (yellow), and the grouping of potential hybrid accessions from northern Queensland.

### 3.5 Genetic variation and structure within *Themeda*

**FIGURE 3:**
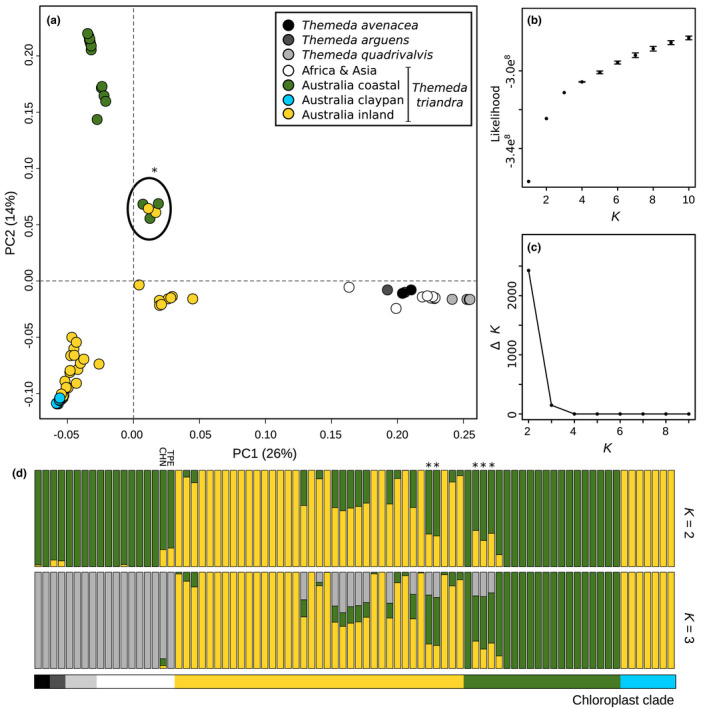
Nuclear genetic variation and structure within *Themeda*. (a) a principal component analysis across the first two axes is shown, with genetic groups coloured based on the chloroplast phylogeny shown in Figure 1. (b) the mean likelihood and standard error for a range of *K*'s is shown, with these values used to calculate Δ*K* (c) as in Evanno et al. (2005). (d) the assignment to genetic clusters is shown for two values of *K*. samples are arranged within their chloroplast clade (indicated by that bar underneath the admixture plots), and ordered from west to east within each group. The asterisks indicate samples with a high degree of admixture from northern Queensland, and accessions from the Philippines (PHI) and China (CHN) are also indicated on the admixture plot.

Genome‐wide genotype likelihoods were used for the population genomics analyses. The principal component analysis largely recovered the nuclear phylogeny groupings, in particular with the clustering of the claypan accessions (blue) with the inland ecotype (yellow; Figure 3a). The first principal component axis explains 26% of the variation in the data and predominantly splits the Australian *T. triandra* samples from the African and Asian *T. triandra* and other outgroup species. The second principal component explains 14% of the variation in the data and splits the Australian accessions into the two distinct nuclear clades, with a small intermediate group of five accessions from northern Queensland. The distinction of Australian *T. triandra* accessions in the PCA is likely to be a direct result of the high proportion (79%) of samples these accessions comprise. We therefore repeated the analysis, only including Australian *T. triandra* accessions, with similar results (Figure S9A). With the Australian *T. triandra* data set PC1 accounts for 21% of variation and it has a significant correlation with sample longitude (Pearson's *r* = 0.63; *p*‐value < .001).

The optimal number of genetic clusters (*K*) based on the admixture analysis of all samples is two, with a secondary optimum of three (Figure 3c). For *K* = 2 the admixture analysis largely distinguishes the Pilbara and inland Australian *T. triandra* accessions from everything else. For *K* = 3 this large mixed grouping is subdivided into the coastal Australian *T. triandra* and all other accessions, including non‐Australian *T. triandra* and other *Themeda* species (Figure 3d). From the admixture analysis alone little can be inferred about the relationships outside of Australia and more broadly across the genus, although the Chinese and Filipino accessions have 15%–20% assignment to the other genetic cluster at *K* = 2, which could indicate possible introgression between Asia and Australia (Figure 3d). This is further supported by the partial assignment of several Australian *T. triandra* accessions to the cluster containing the Asian accessions at *K* = 3.

For Australian *T. triandra* accessions, the most optimal *K* = 2 largely confirms the nuclear phylogeny, with the Pilbara claypan accessions having a mis‐match between their chloroplast and nuclear genomes. The samples are ordered from west to east in each chloroplast genotype block, indicating that there is increased admixture where the two clades come into contact (Figure 3d). There is an extremely high degree of admixture in five individuals with roughly equal proportions of their nuclear genome assigned to Clade I and Clade II (indicated by an asterix in Figure 3d). These are the same individuals from northern Queensland which were intermediate in the PCA (Figure 3a). These individuals also make up the small nuclear clade with mixed chloroplast genotypes in Figure 2. The secondary optimum *K* = 3 largely recovers the same pattern among Australian *T. triandra*. Finally, the same groupings are recaptured when repeating the admixture analysis with only Australian *T. triandra samples* (optimum *K* = 2; secondary optimum *K* = 3; Figure S9).

**FIGURE 4:**
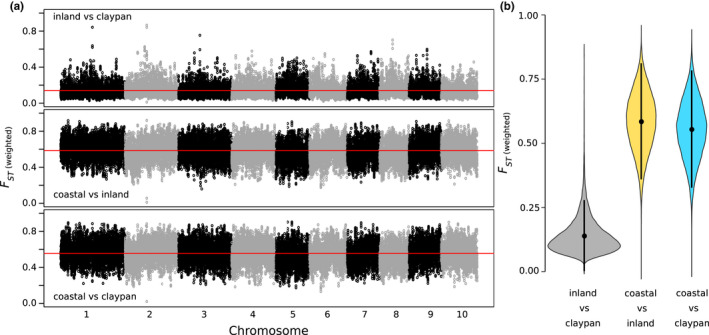
Distribution of *F*_*ST*_ between the three Australian *Themeda triandra* ecotypes/clades considered in this study (coastal, inland and claypan). (a) *F*_*ST*_ values were calculated along the 10 chromosomes in 50 kb windows (10 kb slide), with the red line indicating the mean *F*_*ST*_ value. (b) a violin plot summarizes the *F*_*ST*_ values for each comparison with mean and standard deviation shown.

Pairwise *F*_ST_ confirmed that the nuclear genome of the claypan form and inland ecotype are more similar (*F*_ST_ unweighted = 0.03; *F*_ST_ weighted = 0.15) to each other than to the coastal ecotype (*F*_ST_ unweighted = 0.11; *F*_ST_ weighted = 0.61; and *F*_ST_ unweighted = 0.07; *F*_ST_ weighted = 0.58, respectively). Pairwise *F*_ST_ across the genome showed a similar pattern with no obvious peaks of differentiation (Figure 4).

### 3.6 Introgression in *Themeda*

The admixture analysis did not detect any signs of potential introgression among *T. triandra* and *T. quadrivalvis*, but this could be due to a lack of resolution for the under‐represented outgroup taxa. However, the *D*‐statistic results indicate that there is potential gene flow between Asian *T. triandra* and *T. quadrivalvis*. In every comparison involving an Australian *T. triandra* (Figure S10A), the Asian *T. triandra* had a significant *D*‐statistic indicating introgression with *T. quadrivalvis*, the highest of which involved the Taiwanese accession and was 0.103 (mean = 0.066, SD = 0.012; Figure S10B), with up to 6.9% of the genome being introgressed based on the f4‐ratio (mean = 0.044, SD = 0.008; Figure S10C). When testing for introgression between solely the Australian *T. triandra* accessions and *T. quadrivalvis* there was low‐level introgression detected (maximum *D*‐statistic = 0.036; maximum f4‐ratio = 0.019), but this was uniformly distributed among accessions regardless of distance between the *T. triandra* and *T. quadrivalvis* accessions considered. This probably indicates no ongoing or recent gene flow between *T. triandra* and *T. quadrivalvis* in Australia (Figure S10D).

The *D*‐statistic supported the admixture analysis inference of gene flow between Asian and Australian *T. triandra* (Figure S11), and between the Australian nuclear clades I and II (Figure S12). High levels of admixture were detected between the Taiwanese and Australian *T. triandra* accessions (mean *D*‐statistic = 0.058, SD = 0.038, max = 0.159), particularly in accessions from the north of Australia (Figure S11). Within Australia there was a high degree of introgression (maximum *D*‐statistic = 0.128; maximum f4‐ratio = 0.134), particularly among the accessions identified as having mixed ancestry in the admixture analysis (the highest 135 *D*‐statistic results involved individuals from northern Queensland previously identified as potential hybrids [Figure 3; Table S2]).

### 3.7 Polyploidy is restricted to the inland ecotype

All outgroups and non‐Australian *T. triandra* accessions were assigned as diploid (Table S3). All accessions of the coastal ecotype (green clade in Figure 1) sampled in this study were assigned as diploid apart from two where the ploidy level was unclear (Table S3). No diploids were assigned in the claypan population (blue clade in Figure 1), with a majority (*n* = 6) classified as polyploid and one sample not assigned. Finally, in the inland clade (yellow clade in Figure 1) a majority were assigned as polyploid (56.8%), along with diploid (29.7%) and unassigned accessions (13.5%). There was a lack of resolution within the polyploid assignments, and no accession was assigned as tetraploid, even though previous sampling in southeastern Australia indicated that the tetraploid cytotype was the most common ploidal level for the inland ecotype (Ahrens et al., 2020; Godfree et al., 2017). The phylogenetic placement of the polyploids (Figure S13) and admixture analyses (Figure S9D) hints that they are recent auto‐polyploids, a conclusion supported by previous research showing tetraploids are more closely related to diploids from the same population that those of the same ploidy level elsewhere (Ahrens et al., 2020). All except one of the introgressed individuals of both ecotypes from northern Queensland were confidently assigned as diploid (supported by at least 95.6% of windows), indicating that these were not allopolyploids and probably represent early‐generation hybrids.

### 3.8 Positive selection in a single chloroplast gene

We looked at sequence variation within all 75 protein coding chloroplast genes to detect signs of positive selection between the two Australian *T. triandra* clades potentially involved in a chloroplast capture scenario (i.e., blue and yellow clades; Figure 1) using the ratio of synonymous to nonsynonymous nucleotide substitutions (*ω*). Purifying selection is indicated by *ω* < 1, whereas *ω* = 1 implies neutral evolution and *ω* > 1 indicates positive selection. Out of the 75 genes, 60 had no fixed differences, 11 had fixed differences but they were all synonymous mutations, and four (*ndhf*, *rpl22*, *rpoA*, and *rpoC2*) contained nonsynonymous mutations. Only *rpoC2* had more than three fixed differences and was therefore used for positive selection analysis. A simple pairwise comparison between sequences using the Yang and Nielsen (2000) method indicates *rpoC2* had been under positive selection between the Australian clades, with an excess of nonsynonymous mutations (*ω* = 1.39; dN = 0.0012; dS = 0.0009). This conclusion is supported when testing for positive selection using more complex site models across the phylogeny (M2a > M1a; 2ΔL = 19.60; *p*‐value < .001), which identified one site with >95% probability as being under positive selection (site 1503; *p* = 98.9%). The encoded amino acid at this site is divergent between the clades of interest as a result of a nonsynonymous mutation that has arisen on the branch separating the Chinese/Australian Clade II samples from the Tanzanian accession (Figure 1). However, branch‐site models did not find any significant evidence for elevated positive selection on the branches separating the two clades of interest compared to the rest of the tree (Table S4).

## 4 DISCUSSION

*Themeda triandra* is a particularly interesting tropical grass species. Despite being relatively young (median *T. triandra* crown age 1.48 Ma, 95% HPD range 0.78–3.45; Dunning et al., 2017), this species has become dominant in many African, Asian and Australian grasslands, and has even been dubbed the “The food of the Serengeti grazers” (Sage, 2017). It is the most widely distributed plant species in Australia (Gallagher, 2016) with two predominant ecotypes largely restricted to the wetter, cooler coastal regions or the drier, hotter interior. It is therefore a great model system to investigate rapid environmental adaptation. Here, we use whole‐genome sequencing from over 80 *Themeda* accessions to show that divergence between the inland and coastal populations pre‐dates the colonization of Australia by *T. triandra* based on the organelle phylogenies (Figure 1), and they are therefore not a result of a recent polyploidisation event. We also provide evidence of contemporary gene‐flow where ecotypes come into contact.

### 4.1 Ecotypes predate the colonization of Australia

This study confirms previous findings that Australia was colonized at least twice by *Themeda triandra* (Dunning et al., 2017). Interestingly, these independent colonisations actually represent the arrival of the two different ecotypes, one that inhabits the cooler and wetter coastal regions and the other that is found in the hotter and drier Australian interior (Figure 1). Previous studies have concluded that the differences between these ecotypes can largely be attributed to recent auto‐polyploidisation events in some of the inland individuals (Ahrens et al., 2020; Godfree et al., 2017). While our results support the previous conclusions that ecotypes do largely segregate by ploidy level (Figure S13), the adaptation to these different environments probably pre‐dates the colonization of Australia and any genome duplication events. This is further supported by the presence of known diploids in inland populations identified here, and in previous studies (Ahrens et al., 2020). Therefore, to understand the colonization of the dryland Australian interior it is essential to consider the ecotype's original diversification in Asia. A recent study of *T. triandra* in Yunnan‐Guizhou Plateau in southwest China also found distinct cool‐ and warm‐adapted lineages that are at least two million years old (Chu et al., 2021). However, the distinct Chinese populations are unlikely to be the source of the two Australian ecotypes as the Chinese samples form a monophyletic group based on chloroplast markers, although this is based on a reduced set of markers and a topology with relatively low support (Chu et al., 2021). Even though both ecotypes probably originated in Asia, further sampling across this continent is required to retrace their precise evolutionary origins.

*Themeda triandra* is also widespread in Africa where it grows in a wide range of climatic regions and exhibits a similar diversity in ploidy levels (Snyman et al., 2013) as found here. Although not currently possible, it would be interesting to compare the Australian results with a similar study in Africa. Potentially the African continent was also colonized by multiple ecotypes, and indeed it is notable that African accessions have a similar phylogenetic pattern, being paraphyletic for the chloroplast genome (placement of Tanzanian accession, Figure 1) and monophyletic for the nuclear genome (Figure 2). Comparisons between Australia and Africa will ultimately show if the broad climatic niche *T. triandra* inhabits on both continents is attributed to ancestral genetic variation or rapid convergent evolution.

Ecotypic divergence in other species has been shown to have a complex genetic background, and ploidy differences can accelerate the accumulation of divergent adaptive genetic variation (Lovell et al., 2021). Potentially polyploidy is having the same effect in *T. triandra*, with the inland ecotype more commonly undergoing genome duplication (Figure S13). It is also likely that there is a complex genetic basis to the ecotype differentiation in *T. triandra* as there are no clear peaks of differentiation in the genome (Figure 4), although this is also likely to be attributed to the relatively long divergence time between ecotypes. This is in contrast to more recently diverged ecotypes where patterns of differentiation are less uniform across the genome (Papadopulos et al., 2021). Indeed, many recently evolved ecotypes are formed as a result of standing genetic variation in the ancestral population meaning that the repeated evolution of ecotypes can occur in a relatively short space of time (Papadopulos et al., 2021), although distinguishing this from adaptive introgression between ecotypes can be difficult (Roda et al., 2017). Occasional gene flow between ecotypes can accelerate climate adaptation through the introgression of adaptive loci (Lovell et al., 2021), but how much of a role this plays in the spread of *T. triandra* across the whole of Australia is currently unknown. Although this may be evidenced by the chloroplast capture event in the inland Pilbara claypan populations which may have increased the water use efficiency of these populations (see below).

### 4.2 Hybridisation between ecotypes

The nuclear phylogenies and population genomics results both show incongruences with the organelle data and indicate that there is hybridisation between ecotypes with ongoing gene flow where they come into contact (Figures 2 and 3 and Figure S12). The highest levels of introgression in our data set were localized in individuals from northern Queensland where diploid accessions of both ecotypes are found in close proximity and which have the appearance of early generation hybrids (Figure 2). Populations at increasing distance from this potential hybrid zone contain successively reduced signs of introgression. *Themeda triandra* only relatively recently colonized Australia (<1.3 Ma; Dunning et al., 2017) and at present it is unclear how stable the hybrid zone in northern Queensland is, and it may represent a promising geographic location to investigate the genetic basis of the two ecotypes, although undetected hybrid zones likely exist in other areas as well. It is also likely that the predominant ploidy differences between ecotypes aid in maintaining their divergence (Olofsson et al., 2021).

Potentially, there is also ongoing gene‐flow from Asia into northern Australia (Figure S11). Gene flow between Australian and Asia might also explain why when restricting the analysis to Australian accessions *K* = 3 appears as a secondary optimum as it is still detecting the signal of introgression into accessions from northern Australia from a nonincluded population. Further sampling, particularly in Southeast Asia, is required to confirm this conclusion.

### 4.3 Chloroplast capture in the Pilbara cracking claypans of Western Australia

The Western Australian cracking claypans around Pilbara are characterized by frequent inundation with fresh water, compared to the surrounding drier desert regions. *Themeda triandra* accessions in these restricted habitats have been previously classified as a separate species (*Themeda* sp. Hamersley Station) based on morphological differences, although subsequent inspection by taxonomists have shown these differences are largely qualitative (S. Dillon, personal communication, March 2021). The genetic data indicate that populations from these areas are indeed *T. triandra* (Figures 1 and 2). However, there is clear nuclear and chloroplast discordance in these accessions indicating that the Pilbara population evolved within Australia as a result of adaptive divergence and chloroplast capture. A comparison of the coding genes in the chloroplast indicates that one gene (*rpoC2*) in particular has an ω > 1 indicating potential positive selection in one of the ecotypes. This may mean that the observed chloroplast capture was a result of adaptive introgression.

The *rpoC2* gene encodes a DNA‐dependent RNA polymerase, and its expression has been previously associated with increased water use efficiency in the common bean (*Phaseolus vulgaris*; Ruiz‐Nieto et al., 2015). Comparative analysis of rice chloroplast genomes has also shown *rpoC2* to be under positive selection in *Oryza* species from high light environments, and in particular *Oryza autraliensis*, a wild rice native to northern Australia (Gao et al., 2019). However, when attempting to determine if the amino acid substitutions are convergent between *O. australiensis* and *T. triandra* it became clear that the positive selection result in the former is a likely a false‐positive driven by poorly aligned indel regions rather than actual amino acid substitutions. The role of *rpoC2* in water use efficiency, and the difference in water availability in the cracking claypans versus the surrounding habitat, potentially indicates that adaptive chloroplast capture of the coastal chloroplast has occurred. This pattern might also provide support for a once widespread coastal ecotype across Australia, including its interior, which has then been largely replaced by the interior ecotype as the continent oscillated in water availability before trending to become drier in the last 350,000 years (Kershaw et al., 2003).

### 4.4 Recent speciation of *Themeda quadrivalvis*

Whether *T. quadrivalvis* is a synonym of *T. triandra*, or if it is a separate species has been debated for some time (Keir & Vogler, 2006; Veldkamp, 2016; Dunning et al., 2017; Arthan et al., 2021). *Themeda quadrivalvis* is a globally distributed invasive weed and the only apparent fixed difference between the species is that *T. quadrivalvis* is annual whereas *T. triandra* is perennial. This is the first study to sequence multiple genomes of *T. quadrivalvis* and the results support a previous conclusion that this species has only recently diverged from *T. triandra*. In the early stages of speciation, a daughter species would sit within the larger paraphyletic parental species (Pennington & Lavin, 2016). This is exactly what we observed in the slower evolving chloroplast genome (Figure 1), whereas each species is monophyletic in the nuclear genome (Figure 2). Despite occurring in the same geographic location within Australia, we failed to detect any meaningful ongoing gene flow between these putative species (Figures 3 and Figure S10), suggesting that they are now largely reproductively isolated in Australia and that *T. quadrivalvis* is the product of a recent speciation event. However, we detected introgression between *T. quadrivalvis* and *T. triandra* in Asia, involving up to 6.9% of the nuclear genome (Figure S10). Further work is required to determine if gene flow is ongoing in the native range of *T. quadrivalvis* outside of Australia.

## 5 CONCLUSION

*Themeda triandra* represents one of the most recent and successful rapid radiations of grasses. In a relatively short space of time, it has become the most widely distributed plant species within Australia across a very broad ecological spectrum. Previous research restricted to New South Wales showed that on a relatively local scale adaptation to arid regions can be driven by genome duplication (Ahrens et al., 2020; Godfree et al., 2017). However, on larger continent‐wide scales our research shows that background genetic variation may be more important. Indeed, the ability to occupy almost every climatic niche in Australia is probably a result of independent colonization of the continent by ecotypes within this species, with ploidy variation expanding each of their respective niches. Secondary contact between these ecotypes may further enhance local adaptation by facilitating the introgression of adaptive genetic variation. In summary, the ecotypic differences in *Themeda triandra* appear to be driven by both standing genetic variation and genome duplication, with the importance of either depending on the geographic scale considered.

## AUTHOR CONTRIBUTIONS

Luke T. Dunning and Richard W. Jobson conceived and designed the research with contributions from all coauthors. Paulo C. Baleeiro, Sinethemba Ntshangase, Nigel Barker and Richard W. Jobson conducted fieldwork and generated initial ITS/ETS data. Luke T. Dunning, Samuel G. S. Hibdige, Jill K. Olofsson and Alexander S.T. Papadopulos analysed the whole‐genome data. Oriane Hidalgo and Ilia J. Leitch performed ploidal level and genome size estimations, and Luke T. Dunning and Richard W. Jobson wrote the manuscript and all authors commented on the final version.

## CONFLICT OF INTEREST

The authors declared no conflict of interest for this article.

## Supporting information


Table S1‐S3

Table S4‐Figure S1‐S13

## Data Availability

All raw whole‐genome sequencing data and the TtPh16‐4 reference genome have been deposited with NCBI under Bioproject PRJNA872297. The complete chloroplast genomes have been deposited in NCBI GenBank with accession numbers OP328179–OP328246. Phylogenetic trees and alignments are available from Dryad (reference = https://doi.org/10.5061/dryad.hdr7sqvm9). All scripts used in this study are available on GitHub: https://github.com/Sheffield‐Plant‐Evolutionary‐Genomics/Themeda_triandra‐2022.
