# Supplementary material for: Hybridisation and chloroplast capture between distinct Themeda triandra lineages in Australia
Source: Mol Ecol. 2022 Sep 27;31(22):5846–60. doi: 10.1111/mec.16691 (PMC9828686; doi:10.1111/mec.16691)

MOLECULAR ECOLOGY

**Supplemental Information for:**

# Hybridisation and chloroplast capture between distinct *Themeda triandra* lineages in Australia

**Luke T. Dunning, Jill K. Olofsson, Alexander S.T. Papadopulos, Samuel GS Hibdige, Oriane Hidalgo, Ilia J. Leitch, Paulo C. Baleeiro, Sinethemba Ntshangase, Nigel Barker, Richard W. Jobson**

## Supplementary Tables

**Table S1**: Sample details. (available as supplementary file)

**Table S2**: Test for introgression results. (available as supplementary file)

**Table S3**: Inferring ploidy level results. Samples are ordered as they are in the nuclear phylogeny (Figure S3). (available as supplementary file)

**Table S4:** Results of the branch-site models used to detect positive selection. See Figure S2 for the topology referred to.

| Model | Likelihood | P-value | Daughter Clade |
|---|---|---|---|
| Null model | -6772.91 | - | - |
| Branch 1 | -6772.91 | 1.00E+00 | Claypan clade |
| Branch 2 | -6772.91 | 1.00E+00 | Australian Clade I |
| Branch 3 | -6772.25 | 2.51E-01 | Australian Clade I + Philippines |
| Branch 4 | -6772.91 | 1.00E+00 | Australian Clade I + Philippines +Thailand |
| Branch 5 | -6772.27 | 2.51E-01 | Australian Clade II + China + Tanzania |
| Branch 6 | -6772.91 | 1.00E+00 | Australian Clade II + China |
| Branch 7 | -6772.24 | 2.57E-01 | Australian Clade II |

## Supplementary Figures

**Figure S1:** Initial survey of *Themeda* diversity in Australia. (a) A maximum parsimony topology is inferred from the ITS/ETS barcode region for 332 accession. A subset of 61 Australian *T. triandra* were subsequently selected for whole genome resequencing to ensure the diversity of Australian genotypes was sampled for this species. (b) the geographic distribution of Australian samples is shown.

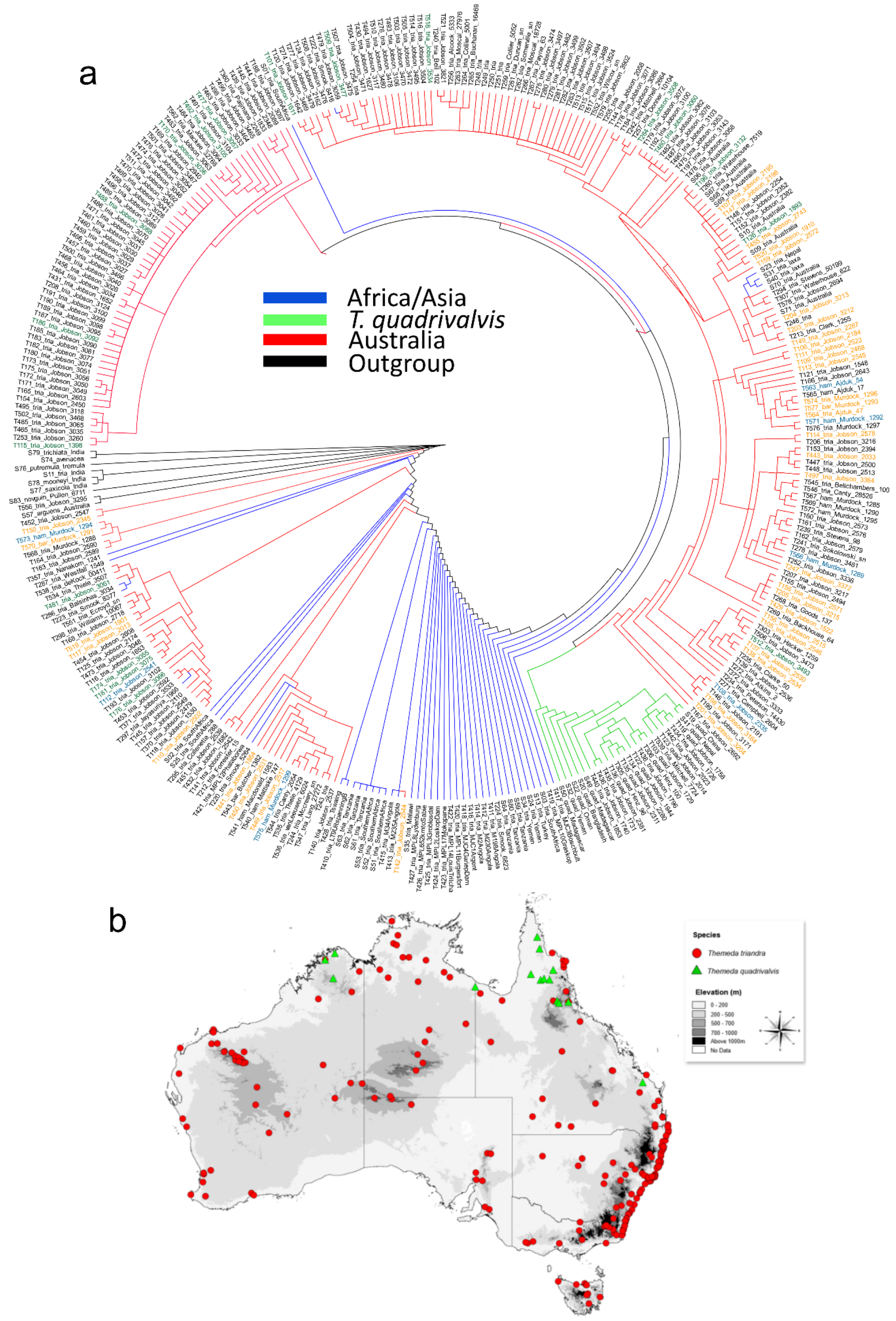

**Figure S2:** Phylogenetic relationships of *Themeda* inferred from whole chloroplast genomes with the inverted repeat removed. The maximum likelihood topology is shown with bootstrap support values based on 100 bootstrap replicates.

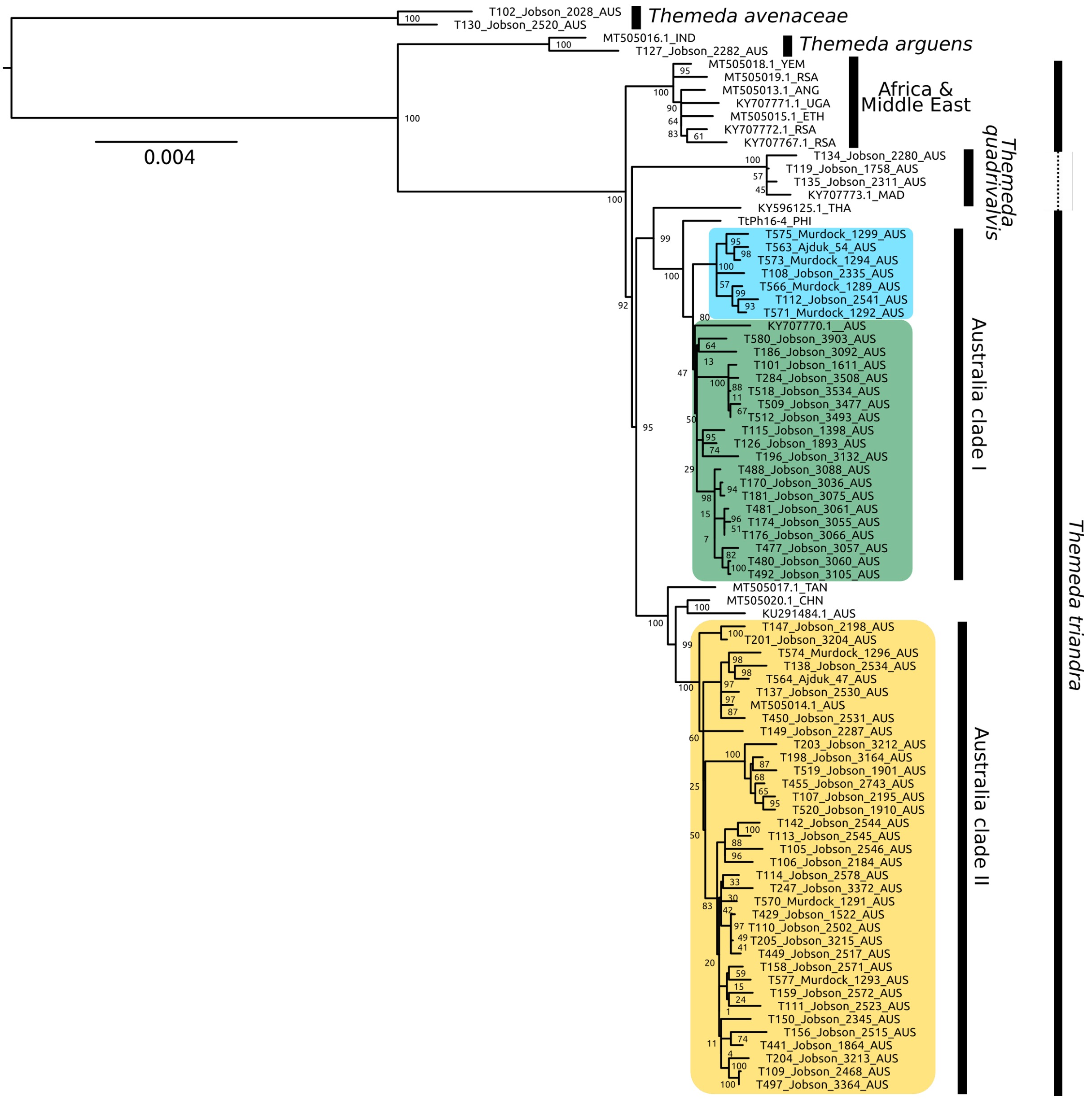

**Figure S3:** Phylogenetic relationships of *Themeda* inferred from the complete chloroplast genome including the inverted repeat. The maximum likelihood topology is shown with bootstrap support values based on 100 bootstrap replicates.

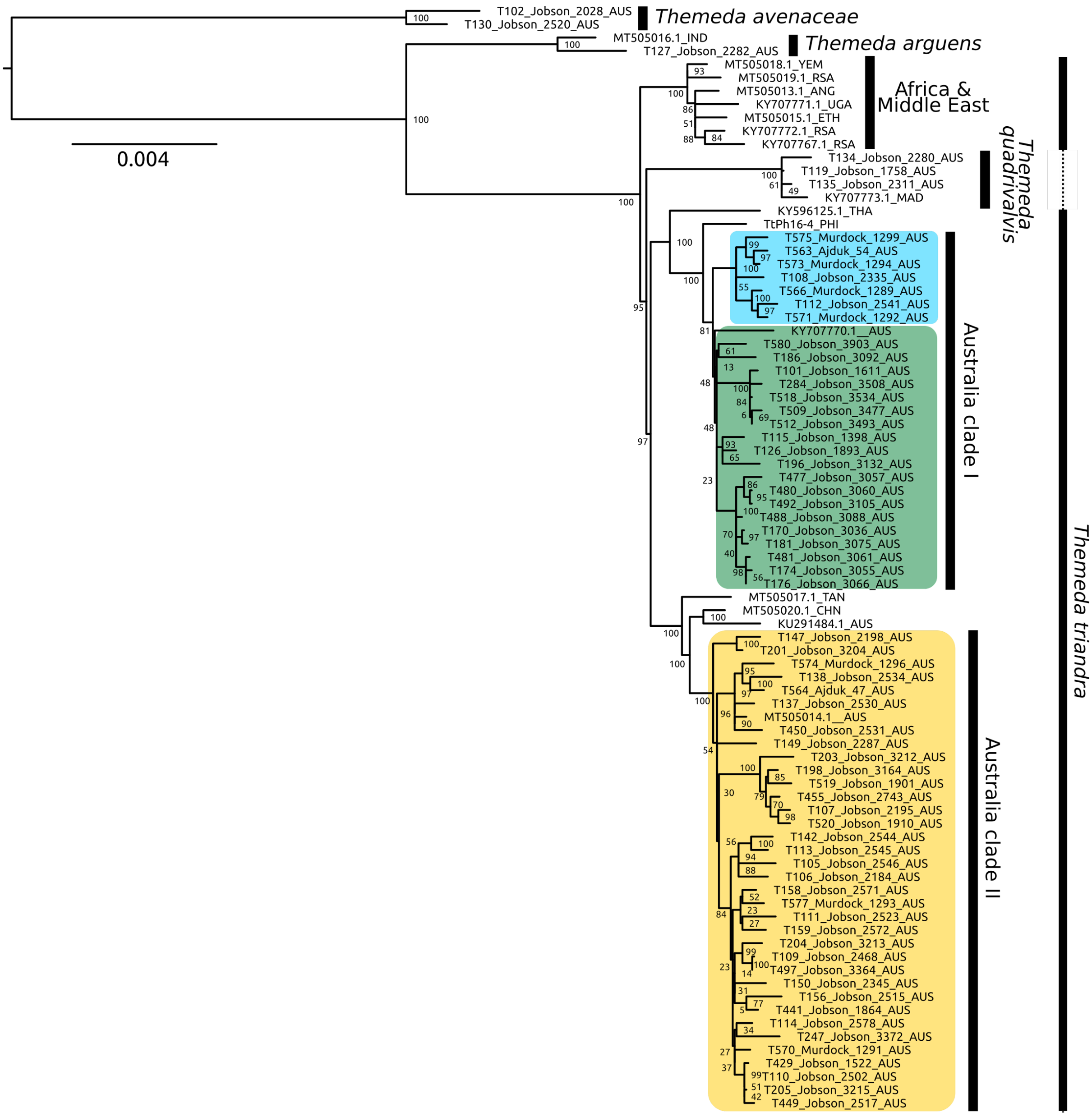

**Figure S4:** Phylogenetic relationships of *Themeda* inferred from the whole mitochondrial genome. The maximum likelihood topology is shown with bootstrap support values.

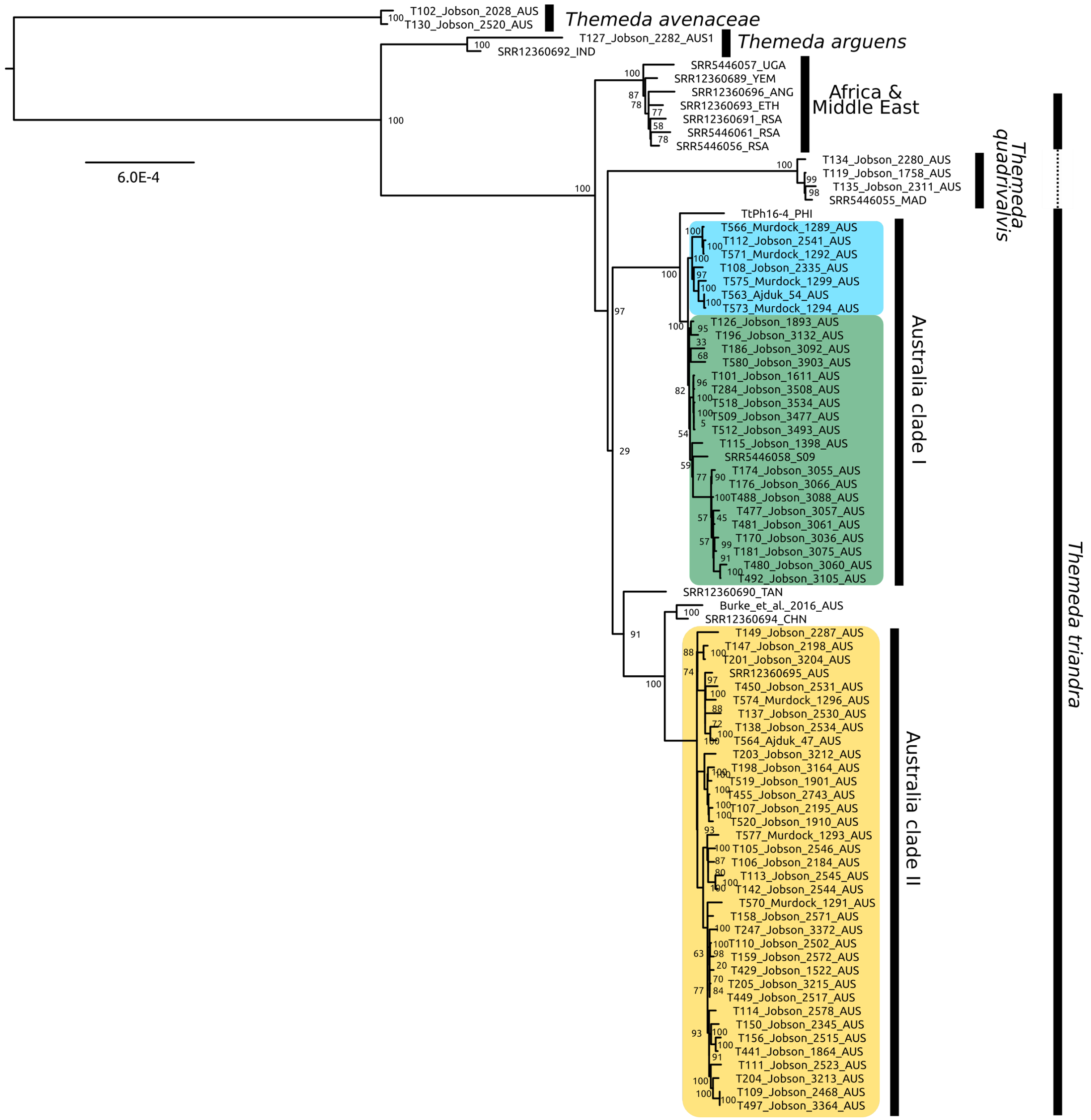

**Figure S5:** Phylogenetic relationships of *Themeda* inferred from a concatenated nuclear alignment of 2,096 genes. The maximum likelihood topology is shown with bootstrap support values based on 1,000 rapid bootstrap replicates. Colours are based on the chloroplast grouping shown in Figure 1.

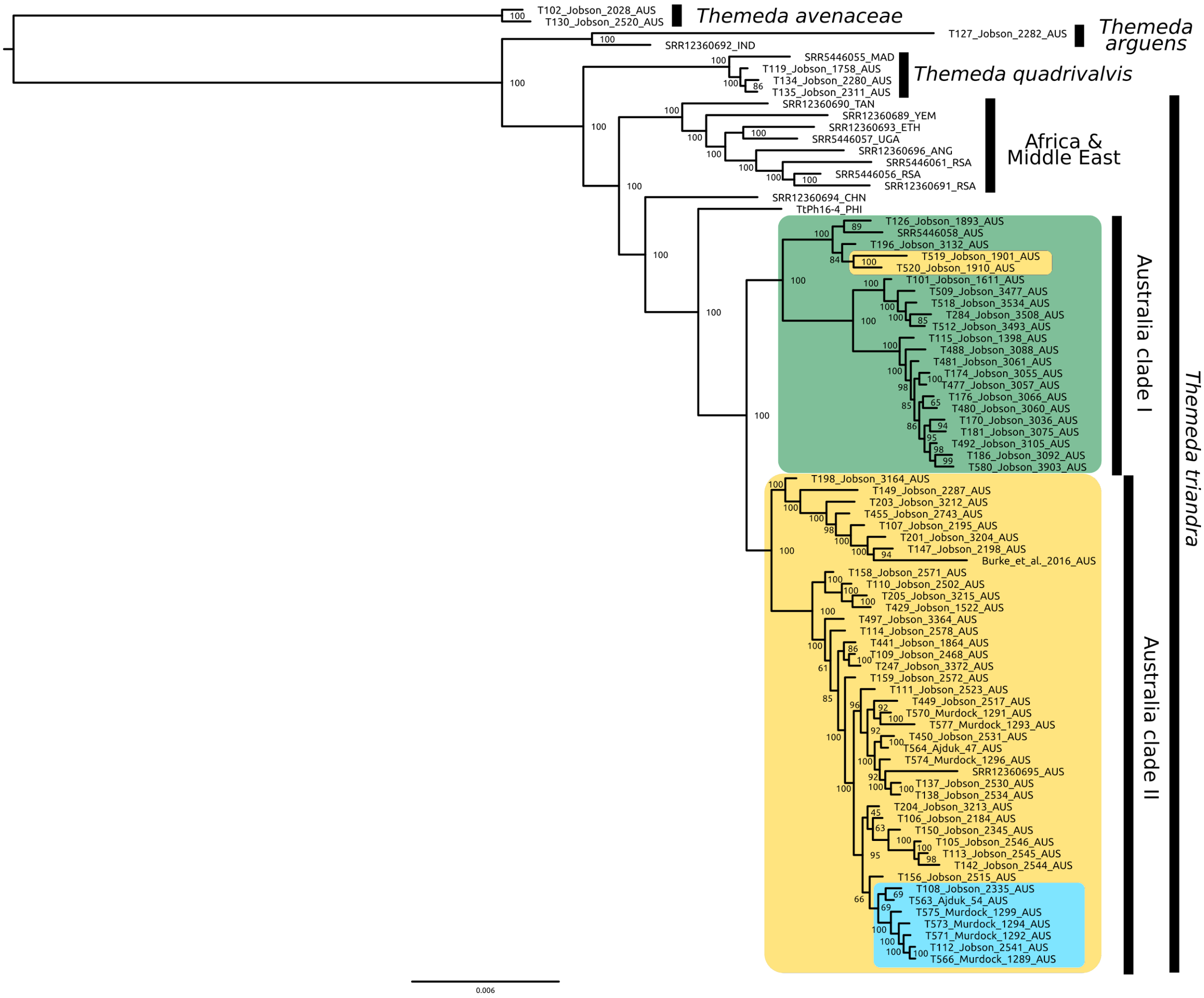

**Figure S6:** Phylogenetic relationships of *Themeda* inferred from 2,096 genes. The coalescence species tree is shown with local posterior probabilities. Branch lengths are in coalescence units, arbitrarily set to one for the tips. Colours are based on the chloroplast grouping shown in Figure 1.

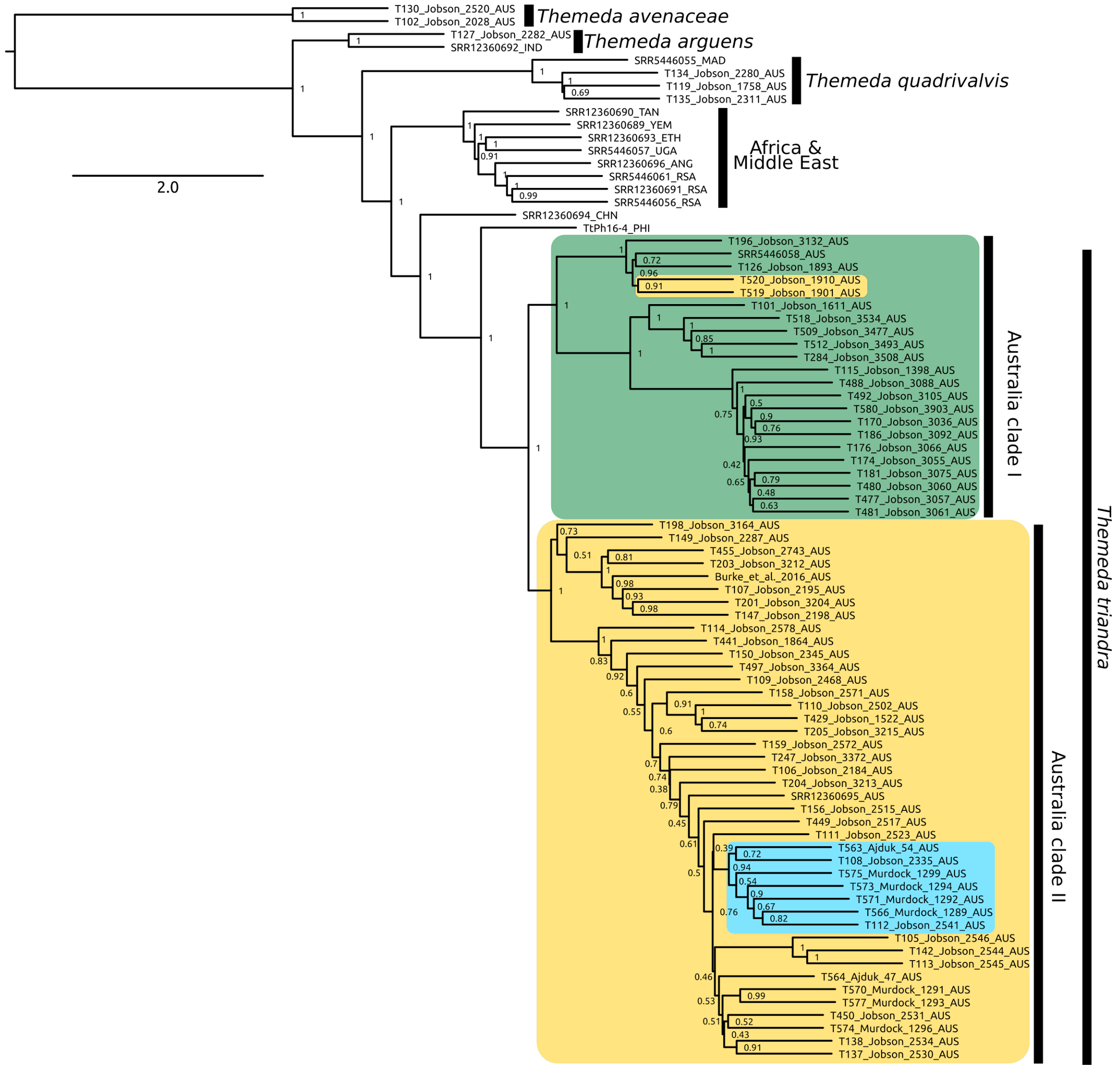

**Figure S7:** Evaluating individual gene tree support for the coalescence species tree topology, with a bootstrap support threshold of either 10% (A) or 50% (B) for each individual gene tree. Terminal Branch lengths are arbitrary. Colours are based on the chloroplast grouping shown in Figure 1.

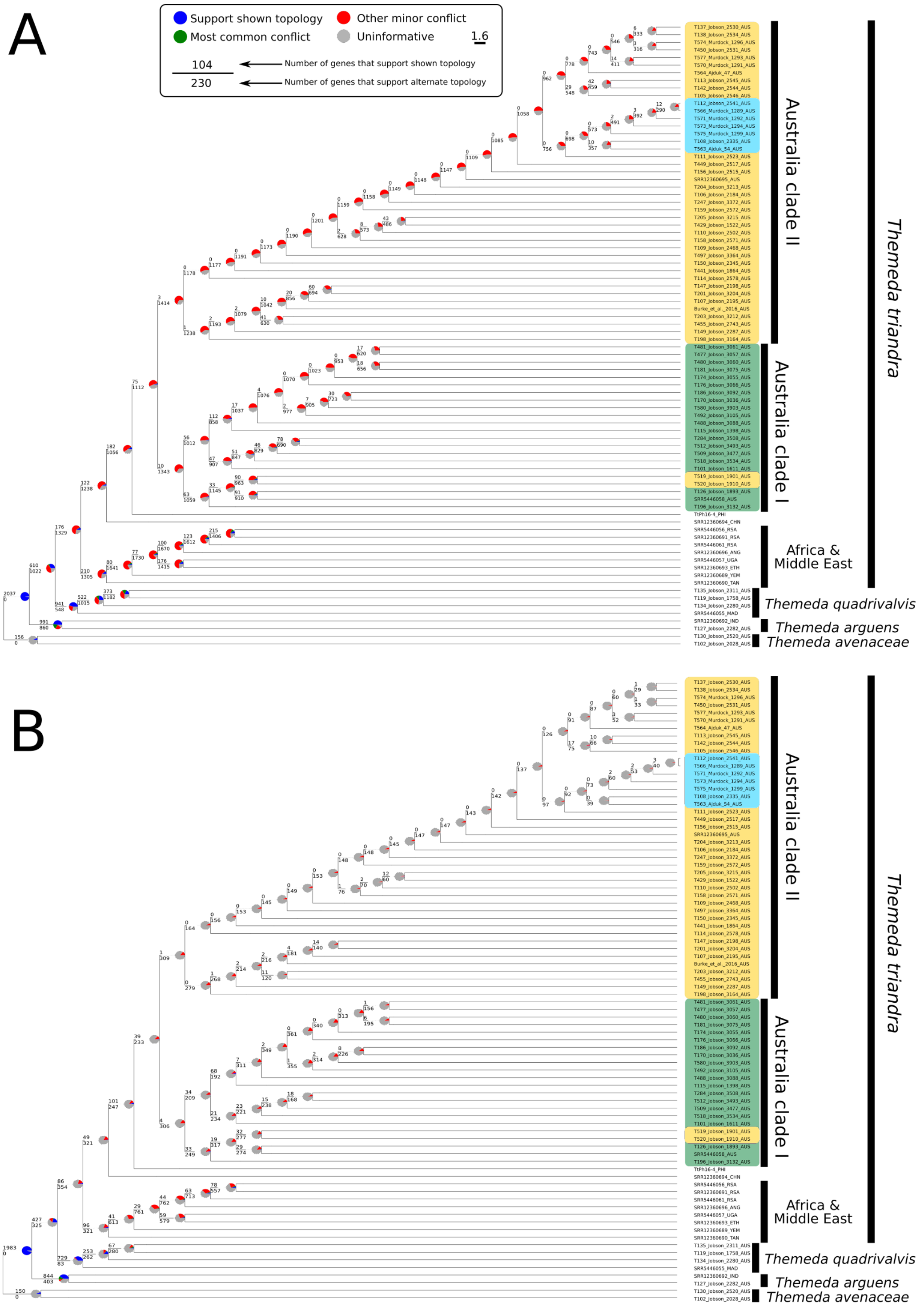

**Figure S8:** Phylogenetic relationships of *Themeda* inferred from the 5.80 kb rDNA coding region consisting of 18S, ITS1, 5.8S, ITS2 and 26S. The maximum likelihood topology is shown with bootstrap support values above 10% indicated. Colours are based on the chloroplast grouping shown in Figure 1. Potential hybrid individuals from northern Queensland are indicated with asterisks.

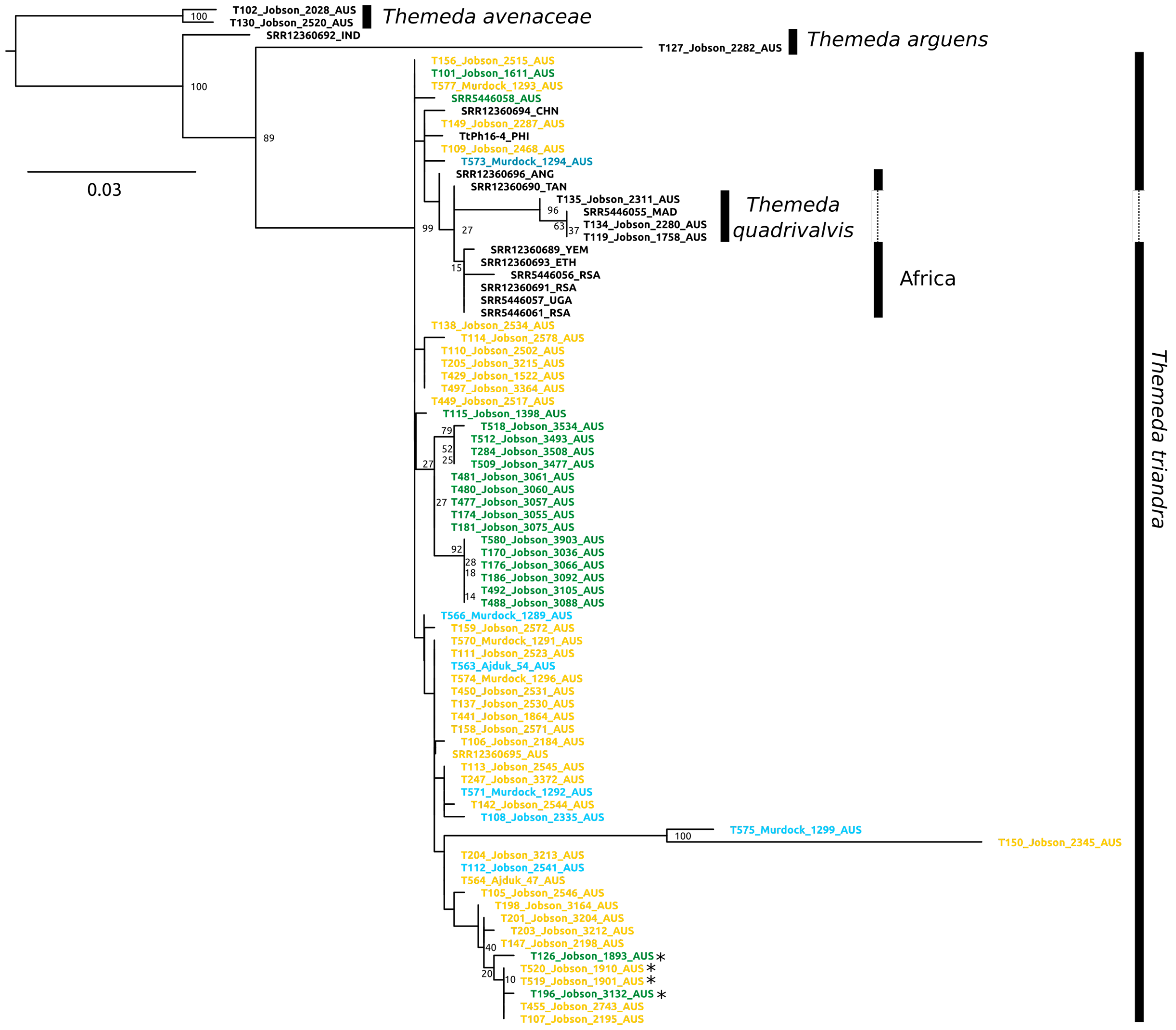

**Figure S9:** Nuclear genetic variation and structure within *Themeda triandra* in Australia. (A) A principal component analysis across the first two axes is shown, with genetic groups coloured based on the chloroplast phylogeny as shown in Figure 1. (B) The mean likelihood and standard deviation for a range of *K*'s is shown, with these values used to calculate Δ*K* (C) as in Evanno et al. (2005). The assignment to genetic clusters is shown for *K* = 2 and *K* = 3. Samples are arranged within their chloroplast clade (indicated by that bar underneath the admixture plots), and ordered from west to east within each group. The asterisks indicate the five samples from northern Queensland with a high degree of admixture.

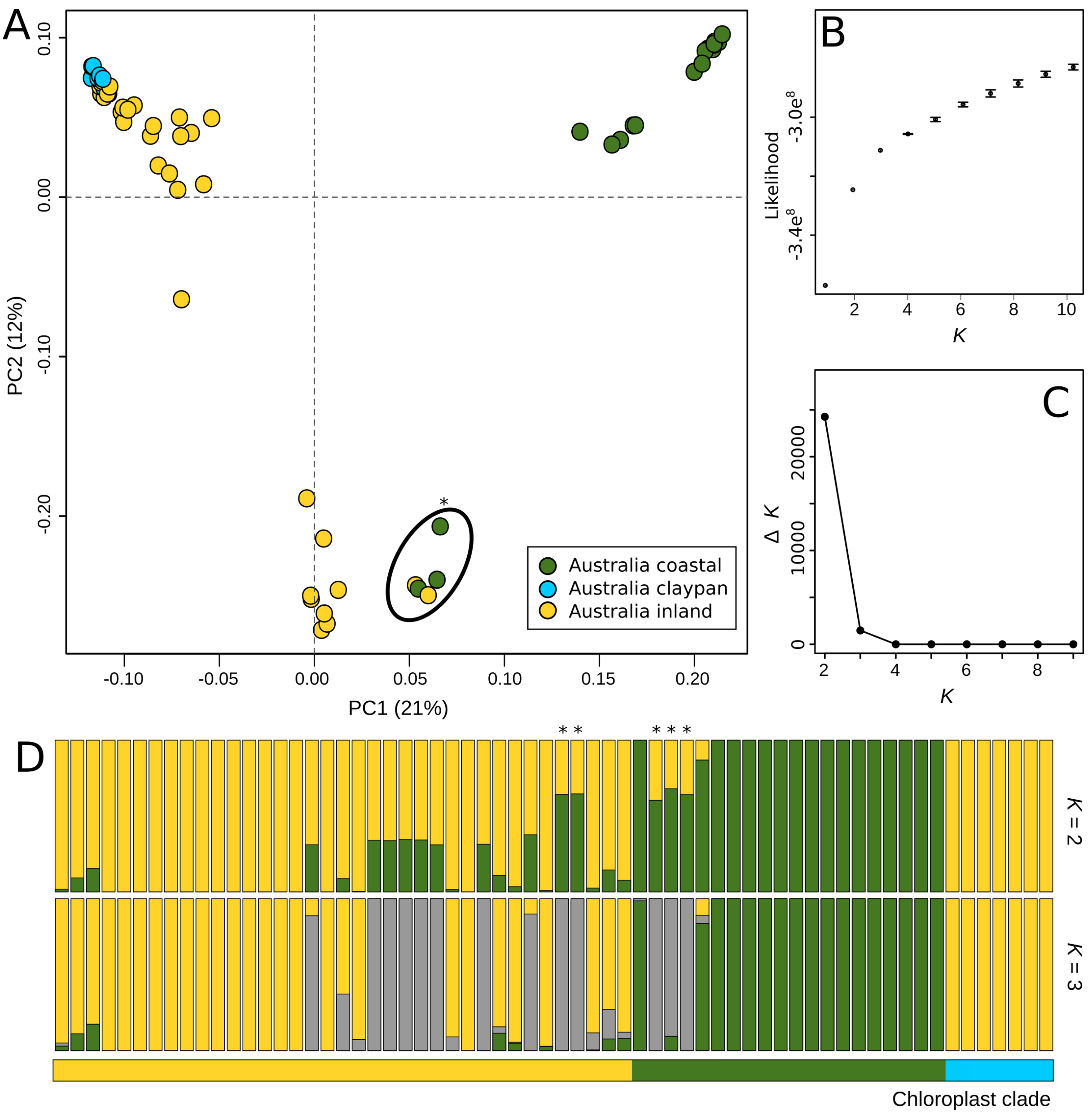

**Figure S10:** Testing for introgression between *Themeda triandra* and *Themeda quadrivalvis*. The scenario tested, *D*-statistic and f4-ratio are shown, with points coloured depending on which sample was in the P2 position. The red arrow in (A) indicates the main conclusion of elevated introgression between *T. quadrivalvis* and Asian *T. triandra* accessions. Summary statistics are based solely on the significant results.

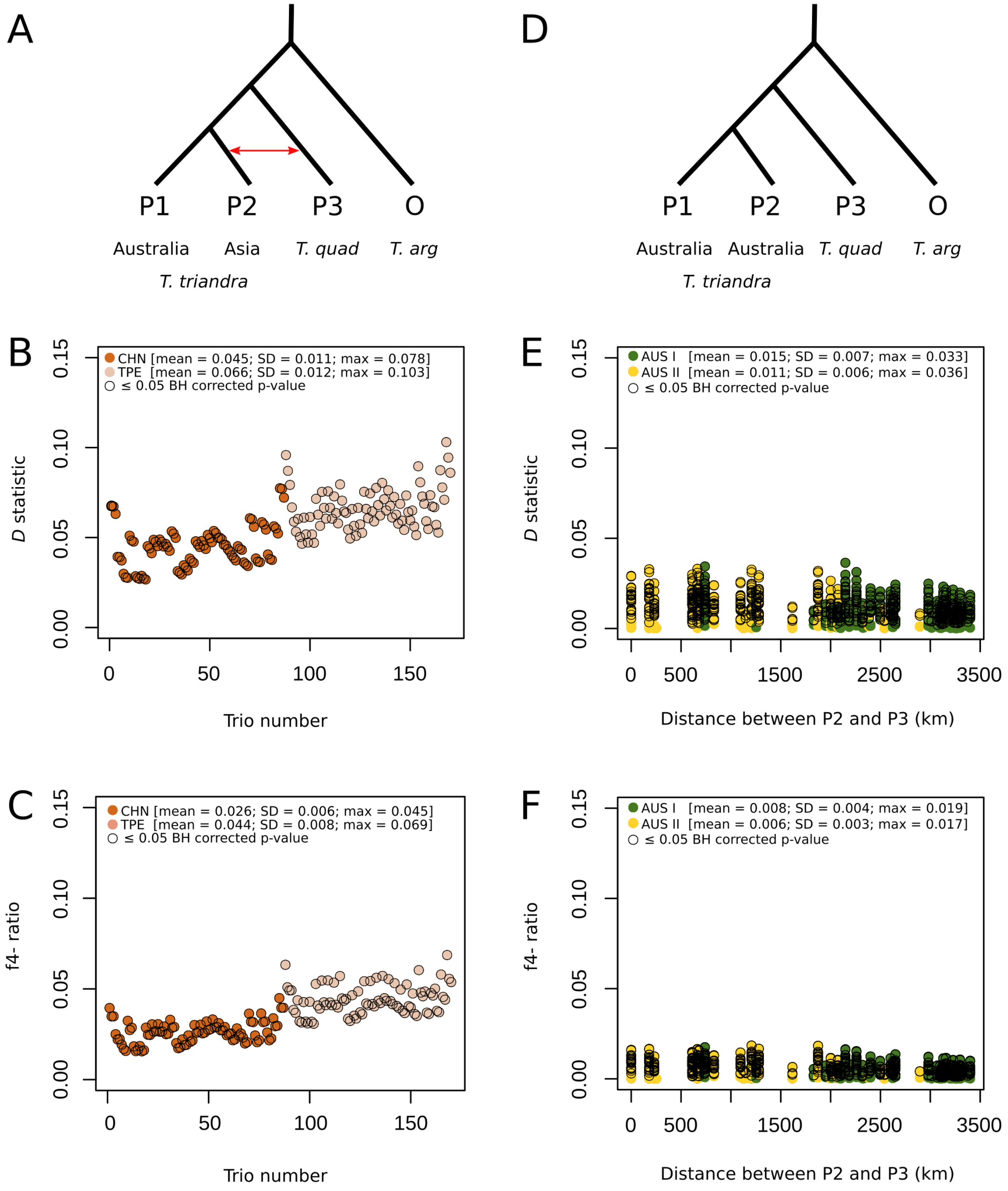

**Figure S11:** Testing for introgression between Australian and Asian *Themeda triandra* Accessions. The scenario tested, *D*-statistic and f4-ratio are shown, with points coloured depending on which sample was in the P3 position. The red arrow in (A) indicates the main conclusion of elevated introgression between the Taiwanese (TPE) and northern Australian accessions. Summary statistics are based solely on the significant results.

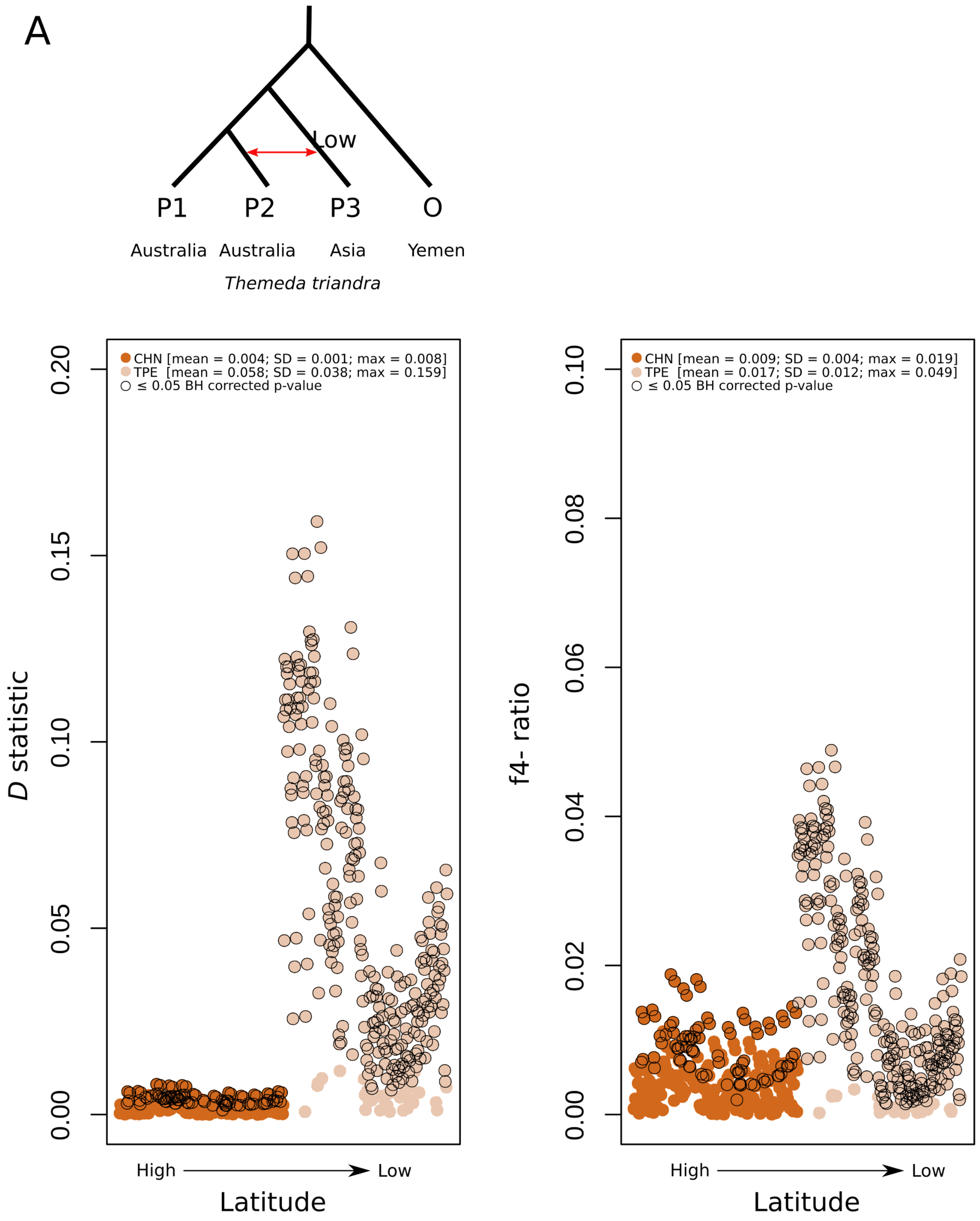

**Figure S12:** Testing for introgression between Australian *Themeda triandra* Accessions. The scenario tested, *D*-statistic and f4-ratio are shown, with points coloured depending on which sample was in the P2 position. The red arrow in (A) indicates the main conclusion that there is introgression between the Australian I and II clades. Summary statistics are based solely on the significant results.

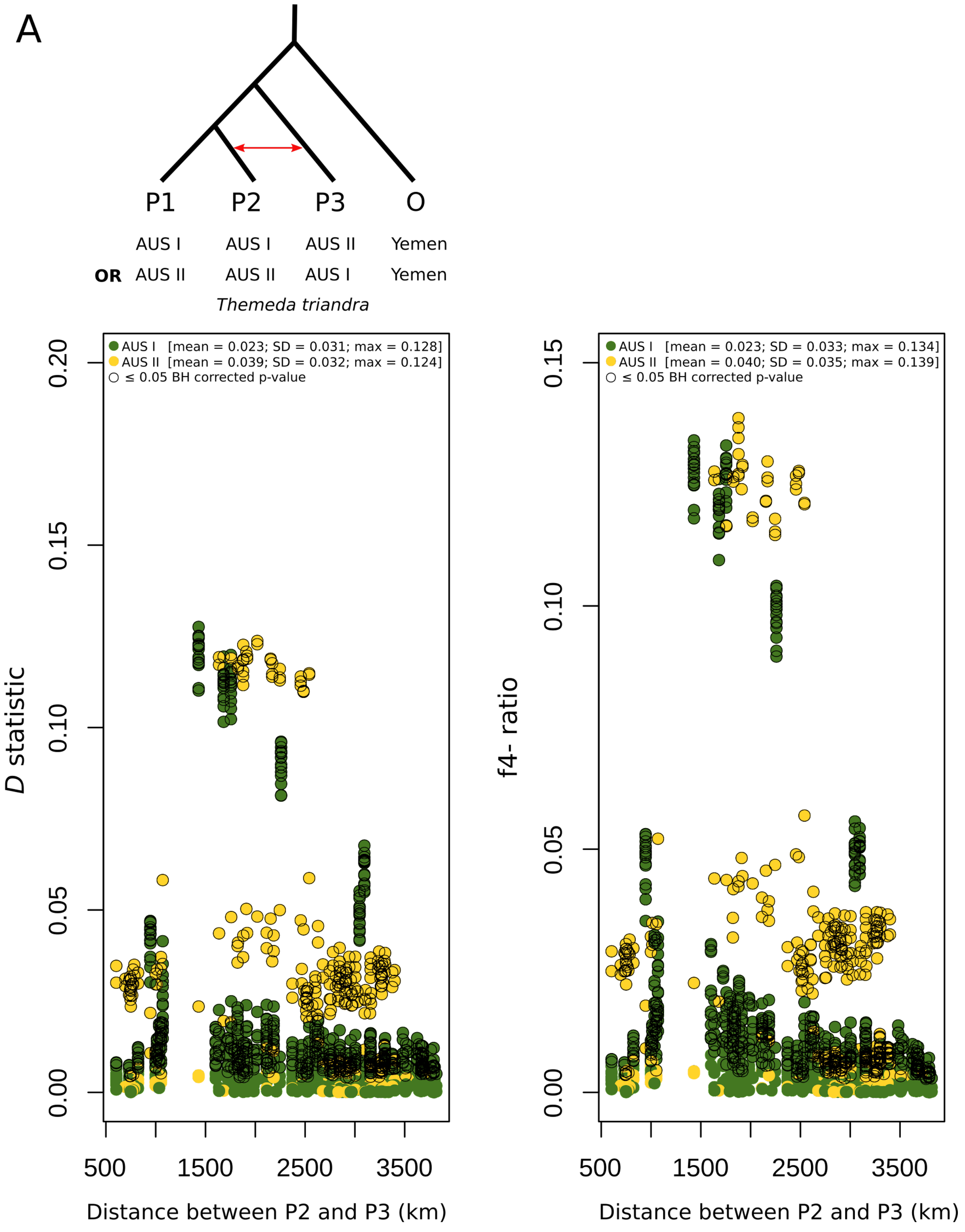

**Figure S13:** Ploidy estimates in *Themeda*. Topology is based on the nuclear concatenated phylogeny (Figures 2A & S5) and the relative number of genomic windows assigned to each ploidy level and the overall ploidy assignment is shown.

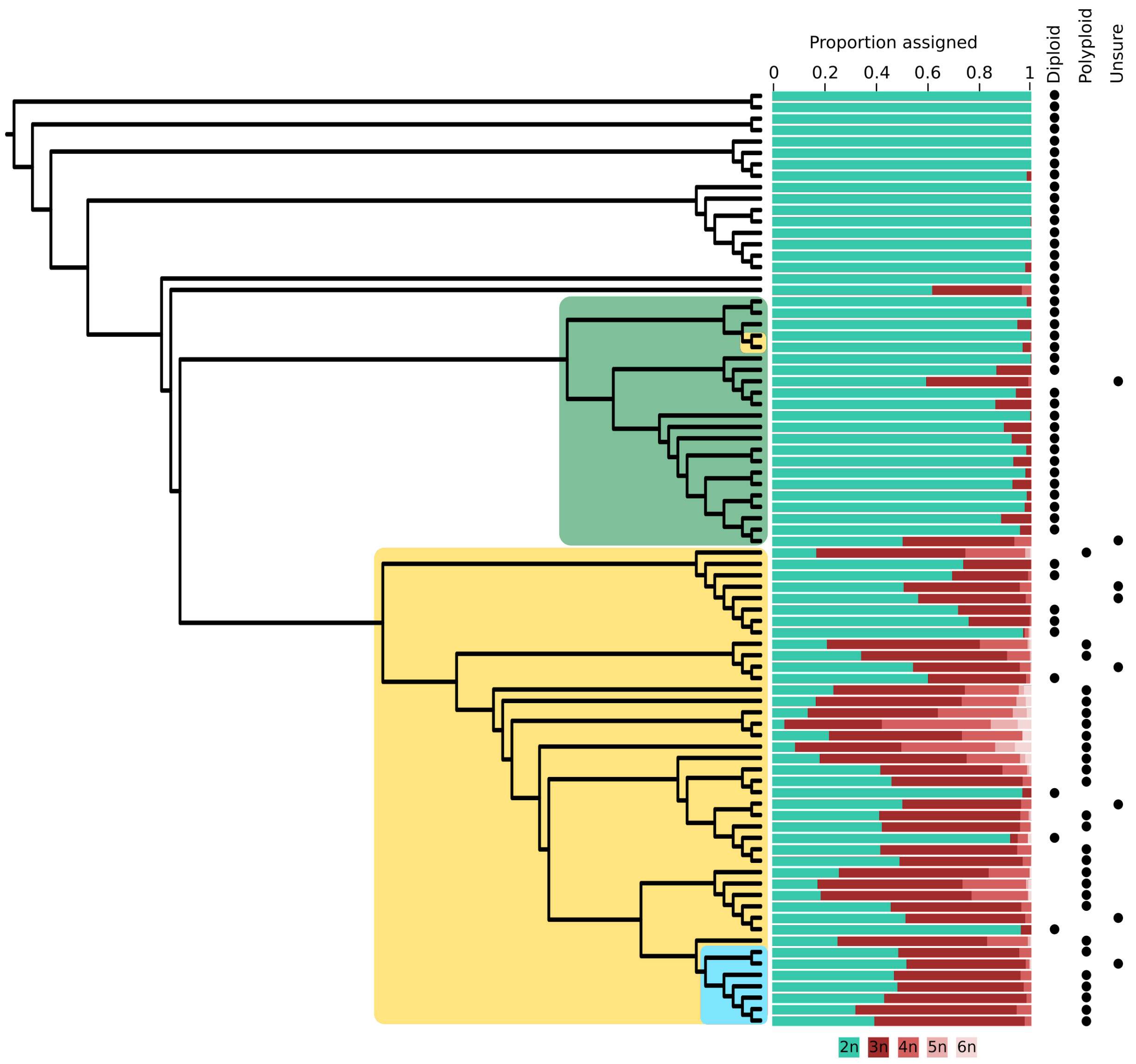

Supplement: Supplementary file 2 — Table S4‐Figure S1‐S13 [file MEC-31-5846-s001.pdf]
